# A Least-Squares Method for the Solution of the Non-smooth Prescribed Jacobian Equation

**DOI:** 10.1007/s10915-022-01968-8

**Published:** 2022-08-24

**Authors:** Alexandre Caboussat, Roland Glowinski, Dimitrios Gourzoulidis

**Affiliations:** 1 Geneva School of Business Administration, University of Applied Sciences and Arts Western Switzerland (HES-SO), Rue de la Tambourine 17, 1227 Carouge, Geneva Switzerland; 2 Institute of Mathematics, Ecole Polytechnique Fédérale de Lausanne (EPFL), 1015 Lausanne, Switzerland; 3 Department of Mathematics, University of Houston, 4800 Calhound Rd, Houston, TX 77204-3008 USA; 4 Hong-Kong Baptist University, Kowloon, Hong Kong

**Keywords:** Jacobian determinant, Least-squares method, Newton methods, Biharmonic regularization, Finite element method, Nonlinear constrained minimization, 65N30, 65K10, 49M20, 35F30

## Abstract

We consider a least-squares/relaxation finite element method for the numerical solution of the prescribed Jacobian equation. We look for its solution via a least-squares approach. We introduce a relaxation algorithm that decouples this least-squares problem into a sequence of local nonlinear problems and variational linear problems. We develop dedicated solvers for the algebraic problems based on Newton’s method and we solve the differential problems using mixed low-order finite elements. Various numerical experiments demonstrate the accuracy, efficiency and the robustness of the proposed method, compared for instance to augmented Lagrangian approaches.

## Introduction

Numerical methods for fully nonlinear equations have recently started to receive a lot of attention, the most well-known equation in that category being the so-called Monge-Ampère equation. Various approaches have been proposed for the numerical solution of second order fully nonlinear equations: the Monge-Ampère equation, see, e.g. [1–6], but also the Pucci’s equation [7–9] or the curvature equation [10, 11].

First order fully nonlinear equations have received slightly less attention: we can mention here the Eikonal equation [12, 13], or the Hamilton-Jacobi equation [14], which have several applications in optics, wave propagation, material science, differential geometry (geodesics), or even economics [15].

The problem of interest here is a particular equation involving the Jacobian of the unknown function, which has been introduced by Dacorogna and Moser (1990) [16]. More precisely, we consider here a Dirichlet boundary value problem inspired by [16–18], which states that, for a given data *f*, we want to find a vector field ${\mathbf {u}}$ satisfying $\det {\nabla {\mathbf {u}}} = f$ in a bounded domain $\Omega \subset {\mathbb {R}}^2$ (together with appropriate Dirichlet boundary conditions). A special interest is paid to problems for which the data *f* is non-smooth. Several works in the literature have focused on this prescribed Jacobian equation, starting with the seminal article [16], which has been developed and extended in, e.g., [17, 19–25]. Existence of classical solutions rely on solutions in Hölder spaces [16]. Weak solutions to the prescribed Jacobian equation must be considered in the sense of Aleksandrov [26], as emphasized, e.g., in [27, 28]. This problem is linked to the Monge-Ampère equation if considering the case where the vector function ${\mathbf {u}}$ is the gradient of a scalar function $\varphi$, as $\det {\nabla {\mathbf {u}}}$ becomes $\det {{\mathbf {D}}^2 \varphi }$. Another related problem in incompressible elasticity has been addressed in [29] (see also the references therein), where the incompressibility condition reads as $\mathrm {det}({\mathbf {I}}_d +\nabla {\mathbf {u}}) =1$, ${\mathbf {I}}_d$ being the identity tensor and ${\mathbf {u}}$ the unknown displacement field.

Theoretical solutions, obtained with explicit constructions, exist in the literature for cases in simple geometries, and usually with identity boundary conditions. However, numerical methods for this particular problem are rather scarce. The goal of the present article is to design a numerical method for the finite element approximation of the prescribed Jacobian equation for arbitrary two-dimensional domains, including non-convex domains and non-smooth data.

A first methodology has been proposed in [30, 31] by using an *Alternating Direction Method of Multipliers* (ADMM) approach. The numerical algorithm has proved to be efficient, but requires a tedious fine tuning of parameters.

Following previous works on the Monge-Ampère equation [1, 32], we advocate here a variational approach for the solution of the prescribed Jacobian equation that is based on a least-squares approach. In order to decouple the nonlinearities of the problem from the linear variational aspects, we use a relaxation algorithm. Low order finite elements are used for the space discretization, while mathematical programming methods [33] allow to solve local constrained optimization problems. Equality constraints are taken into consideration via a Lagrangian approach.

In order to demonstrate the flexibility of the proposed method, we also consider in addition the *prescribed Jacobian inequality*. The, strongly underdertemined, problem consists in finding a vector field ${\mathbf {u}}$ satisfying $\det {\nabla {\mathbf {u}}} \ge f$ instead of $\det {\nabla {\mathbf {u}}} = f$ (together with appropriate Dirichlet boundary conditions). Theoretical results of the prescribed Jacobian inequality are given, e.g., in [34], and to the best of our knowledge there are no proposed numerical methods to solve this type of inequality. It will be addressed by, e.g., including interior-point methods [33].

The numerical validation is achieved first via the solution of test problems allowing a computational investigation of the convergence properties of our methodology. The treatment of more demanding test problems associated with non-smooth data and/or non-convex domains, allows to investigate the accuracy and the robustness of the proposed methodology.

## The Mathematical Problem

Let $\Omega$ be a bounded domain of ${\mathbb {R}}^2$. We denote by $\Gamma$ the boundary of $\Omega$. Let $f : \Omega \rightarrow {\mathbb {R}}$ and ${\mathbf {g}}: \Gamma \rightarrow {\mathbb {R}}^2$ be given sufficiently smooth functions. The partial differential equation involving the Jacobian determinant we want to solve reads as follows: find ${\mathbf {u}}: \Omega \rightarrow {\mathbb {R}}^2$ satisfying

1 $$\begin{aligned} \left\{ \begin{array}{ll} \det {\nabla {\mathbf {u}}} = f &{} \text { in } \Omega , \\ {\mathbf {u}}= {\mathbf {g}}&{} \text { on } \Gamma . \end{array} \right. \end{aligned}$$

Generally, we assume that $f\ge 0$. This problem has been originally investigated in [16] from a theoretical point of view, when the boundary condition is the identity function (i.e. ${\mathbf {g}}\left( {\mathbf {x}}\right) = {\mathbf {x}}$ for ${\mathbf {x}}\in \Gamma$). The corresponding problem is of the following type:

2 $$\begin{aligned} \left\{ \begin{array}{ll} \det {\nabla {\mathbf {u}}} = f &{} \text {in } \Omega , \\ {\mathbf {u}}\left( {\mathbf {x}}\right) = {\mathbf {x}}&{} a.e. \, {\mathbf {x}}\text { on } \Gamma . \end{array} \right. \end{aligned}$$

Problem (2) corresponds to finding a mapping ${\mathbf {u}}$ that preserves both the boundary data and some kind of volume (up to some stretching of the mapping). These problems have been addressed in [30, 31] with a numerical approach based on augmented Lagrangian techniques. We will provide here an alternative, more robust, numerical method, based on a least-squares approach.

Note that the solution to (2) is not necessarily unique and the same remark holds for (1). Indeed, let us consider (2) with $f = 1$ and $\Omega$ the unit disk centered at the origin; in this case, ${\mathbf {u}}\left( {\mathbf {x}}\right) = {\mathbf {x}}$ is an obvious solution. But, when using the polar coordinates $\left( \rho , \theta \right)$, one can see that ${\mathbf {v}}$ defined by ${\mathbf {v}}\left( \rho ,\theta \right) = \left( \rho \cos \left( \theta + 2k\pi \rho ^2\right) \,,\, \rho \sin \left( \theta + 2k\pi \rho ^2\right) \right) ^T$ is also a solution.

The proof of the existence of a solution to (1) (via the divergence theorem) requires data to be compatible with the geometrical domain, see [16]. When the boundary conditions are given by ${\mathbf {u}}\left( {\mathbf {x}}\right) = {\mathbf {x}}$ on $\Gamma$, this compatibility condition reads as:

3 $$\begin{aligned} \int _\Omega f d{\mathbf {x}}= \, \text { measure} \left( \Omega \right) . \end{aligned}$$

The positiveness of the right-hand side *f* is useful from an analytical point of view to prove existence results. Moreover, it makes problem (1) an elliptic problem, which is important for the solution methodology discussed in this article. However, it has been loosened (slightly) to accept locally negative data (see, e.g., [17]). From now on we will assume that (3) holds.

Originally, the regularity of (classical) solutions in Hölder spaces can be found in [16]. In order to derive such regularity results and obtain classical solutions in $C^{k+1,\alpha }({\bar{\Omega }})$, the regularity needed on the data is $f,g \in C^{k,\alpha }({\bar{\Omega }})$, together with $f,g >0$ and the compatibility condition (3). Moreover, existence results require that the regularity of the domain is $\partial \Omega$ has to be $C^{k+3,\alpha }({\bar{\Omega }})$.

On the other hand, weak solutions to the generated Jacobian equation must be considered in the Aleksandrov-sense [26–28, 35, 36]. The derivation and proofs are similar to the approach for solutions to the Monge-Ampère equation, see, e.g., [2]. As stated earlier, the relationship between the Monge-Ampère equation $\mathrm {det} {\mathbf {D}}^2 \psi = f$ and the Jacobian equation $\mathrm {det} \nabla {\mathbf {u}} = f$ is explicit, by setting ${\mathbf {u}} = \nabla \psi$ [22]. It is thus not surprising to find similar concepts of weak solutions.

The proposed method allows to find a finite element approximation *in a least-squares sense*. As in [1, 32], there is no evidence that the discrete solution is converging to an Aleksandrov-type of solution. However, experiments have exhibited a strong numerical evidence of a convergent behavior of the approximations when the discretization parameters tend to zero.

### Remark 1

(*Jacobian problem with inequalities*) In parallel, we consider the following partial differential inequality : find ${\mathbf {u}}: \Omega \rightarrow {\mathbb {R}}^2$ satisfying

4 $$\begin{aligned} \left\{ \begin{array}{ll} \det {\nabla {\mathbf {u}}} \ge f &{} \text { in } \Omega , \\ {\mathbf {u}}= {\mathbf {g}}&{} \text { on } \Gamma . \end{array} \right. \end{aligned}$$

with again, in particular, the case of the identity boundary condition (i.e. ${\mathbf {g}}\left( {\mathbf {x}}\right) = {\mathbf {x}}$ for ${\mathbf {x}}\in \Gamma$). This problem has been addressed in [34] where existence results are established under the condition

5 $$\begin{aligned} \int _\Omega f d{\mathbf {x}}\le \, \text {measure} \left( \Omega \right) . \end{aligned}$$

We will show in the sequel that the numerical techniques developed for (1) also apply naturally to (4), with small modifications. $\square$

## A Numerical Algorithm

We advocate a numerical algorithm based on a least-squares approach and a relaxation algorithm. The relaxation algorithm allows to split the minimization problem into a sequence of subproblems. The first subproblem consists of low dimensional local nonlinear problems, the number of them being determined from the chosen mesh grid. The second subproblem is a linear variational problem and it results in a fourth-order partial differential equation.

### A Least-Squares Method

Let us define

$$\begin{aligned} {\mathbf {Q}}_f=\left\{ {\mathbf {q}}\in \left( L^2\left( \Omega \right) \right) ^{2 \times 2 }, \text {det}{\mathbf {q}}=f \right\} , \quad {\mathbf {V}}_{\mathbf {g}}=\left\{ {\mathbf {v}}\in \left( H^1 \left( \Omega \right) \right) ^2,{\mathbf {v}}\vert _{\partial \Omega }={\mathbf {g}} \right\} . \end{aligned}$$

We assume that *f* and *g* are sufficiently smooth, so that ${\mathbf {Q}}_f$ and ${\mathbf {V}}_{\mathbf {g}}$ are non-empty. The least squares problem introduces an auxiliary variable ${\mathbf {q}}\in {\mathbf {Q}}_f$, and reads as follows: find $\mathbf {\left\{ {\mathbf {u}},{\mathbf {p}}\right\} }\in {\mathbf {V}}_{\mathbf {g}} \times \ {\mathbf {Q}}_f$ such that

6 $$\begin{aligned} J\left( {\mathbf {u}},{\mathbf {p}}\right) \le J\left( {\mathbf {v}},{\mathbf {q}}\right) ,\ \ \forall \left\{ {\mathbf {v}},{\mathbf {q}}\right\} \in {\mathbf {V}}_{\mathbf {g}}\times {\mathbf {Q}}_f, \end{aligned}$$

where $J(\cdot ,\cdot )$ is defined by

7 $$\begin{aligned} J \left( \mathbf {v,q}\right) =\frac{1}{2}\int _\Omega \left| \nabla {\mathbf {v}}- {\mathbf {q}}\right| ^2 \text {d} {\mathbf {x}}. \end{aligned}$$

Here $\vert \cdot \vert$ denotes the Frobenius norm $\left| {\mathbf {T}}\right| =\left( {\mathbf {T}}: {\mathbf {T}}\right) ^{1/2}$, with the inner product ${\mathbf {S}}: {\mathbf {T}}= \sum _{i,j=1}^2 s_{ij}t_{ij}$ where ${\mathbf {S}},{\mathbf {T}}$ are $2\times 2$ matrices with elements $s_{ij}$, $t_{ij}$ for $i,j=1,2$, respectively.

We propose a biharmonic regularization of the objective function. The added term is motivated by previous works involving first-order elliptic equations, see [12, 31, 37]. The modified objective function is defined as

8 $$\begin{aligned} J_\varepsilon \left( \mathbf {v,q}\right) =J \left( \mathbf {v,q}\right) + \frac{\varepsilon }{2} \int _\Omega \left| \nabla ^2 {\mathbf {v}}\right| ^2dx, \end{aligned}$$

where $\varepsilon \ge 0$. Numerical experiments will illustrate that the biharmonic regularization allows to accelerate the convergence of the numerical algorithm. The modified minimization problem reads as: find $\mathbf {\left\{ {\mathbf {u}},{\mathbf {p}}\right\} }\in {\bar{{\mathbf {V}}}}_{\mathbf {g}} \times \ {\mathbf {Q}}_f$ such that

9 $$\begin{aligned} J_\varepsilon \left( {\mathbf {u}},{\mathbf {p}}\right) \le J_\varepsilon \left( {\mathbf {v}},{\mathbf {q}}\right) ,\ \ \forall \left\{ {\mathbf {v}},{\mathbf {q}}\right\} \in {\bar{{\mathbf {V}}}}_{\mathbf {g}}\times {\mathbf {Q}}_f, \end{aligned}$$

where $${\bar{{\mathbf {V}}}}_{\mathbf {g}}={\mathbf {V}}_{\mathbf {g}}\cap \left( H^2\left( \Omega \right) \right) ^2$$.

#### Remark 2

(*Jacobian problem with inequality*) In the case of the inequality (4), we modify the functional space ${\mathbf {Q}}_f$ as

$$\begin{aligned} {\tilde{{\mathbf {Q}}}}_f=\left\{ {\mathbf {q}}\in \left( L^2\left( \Omega \right) \right) ^{2 \times 2 }, \mathrm {det}\ {\mathbf {q}}\>\ge f \right\} . \end{aligned}$$

The objective function $J_\varepsilon \left( \cdot , \cdot \right)$ remains the same as in (8), and the minimization problem reads as: find $\mathbf {\left\{ {\mathbf {u}},{\mathbf {p}}\right\} }\in {\bar{{\mathbf {V}}}}_{\mathbf {g}} \times \ {\tilde{{\mathbf {Q}}}}_f$ such that

10 $$\begin{aligned} J_\varepsilon \left( {\mathbf {u}},{\mathbf {p}}\right) \le J_\varepsilon \left( {\mathbf {v}},{\mathbf {q}}\right) ,\ \ \forall \left\{ {\mathbf {v}},{\mathbf {q}}\right\} \in {\bar{{\mathbf {V}}}}_{\mathbf {g}}\times {\tilde{{\mathbf {Q}}}}_f. \end{aligned}$$

$$\square $$

### A Relaxation Algorithm

For the solution of (9) and (10) respectively, we propose the following relaxation algorithm:

1. The initial guess of the algorithm is obtained by solving:
  11 $$\begin{aligned} -\nabla ^2 {\mathbf {u}}^1=\tilde{{\mathbf {f}}} \text { in } \Omega , \qquad {\mathbf {u}}^1 = {\mathbf {g}}\text { on } \Gamma , \end{aligned}$$
  where $\tilde{{\mathbf {f}}}=\left( 1,1\right)$. The solution of (11) is smooth, convex, and matches the boundary conditions of (1). Then, for $n\ge 1$:
2. When ${\mathbf {u}}^n$ is known, we look for
  12 $$\begin{aligned} {\mathbf {p}}^{n} = \arg \min _{{\mathbf {q}}\in {\mathbf {Q}}_f} J_\varepsilon \left( {\mathbf {u}}^{n},{\mathbf {q}}\right) ; \end{aligned}$$
3. When ${\mathbf {p}}^n$ is known, we look for
  13 $$\begin{aligned} {\mathbf {u}}^{n+1/2} = \arg \min _{{\mathbf {v}}\in {\bar{{\mathbf {V}}}}_g} J_\varepsilon \left( {\mathbf {v}},{\mathbf {p}}^n\right) ; \end{aligned}$$
4. When ${\mathbf {u}}^{n+1/2}$ is known, we update the solution by
  14 $$\begin{aligned} {\mathbf {u}}^{n+1} = {\mathbf {u}}^{n} + \omega \left( {\mathbf {u}}^{n+1/2} - {\mathbf {u}}^n\right) , \end{aligned}$$
  where $\omega \in \left( 0,2 \right)$ is a relaxation parameter that helps controlling the convergence speed.

#### Remark 3

(*Jacobian problem with inequality*) For the Jacobian inequality (4), the solution can be found by replacing in (12) the functional space ${\mathbf {Q}}_f$ by ${\tilde{{\mathbf {Q}}}}_f$ defined in (10). $\square$

### Numerical Solution of the Local Nonlinear Problems

We focus here on the solution of (12). Since ${\mathbf {u}}^n$ is known, the solution ${\mathbf {p}}^n$ is obtained by solving the minimization problem

15 $$\begin{aligned} {\mathbf {p}}^n = \arg \min _{{\mathbf {q}}\in {\mathbf {Q}}_f} \left[ \int _\Omega \frac{1}{2} \left| {\mathbf {q}}\right| ^2 d{\mathbf {x}}- \int _\Omega \nabla {\mathbf {u}}^{n} : {\mathbf {q}}d{\mathbf {x}}\right] . \end{aligned}$$

Problem (15) can be solved point-wise since it does not involve any derivative for the variable ${\mathbf {q}}$. The solution can be obtained, locally for all ${\mathbf {x}}$ $\in \Omega$, as

16 $$\begin{aligned} {\mathbf {p}}^n\left( {\mathbf {x}}\right) = \arg \min _{{\mathbf {q}}\in {\mathbf {E}}_{f({\mathbf {x}})}} \left[ \frac{1}{2} \left| {\mathbf {q}}\right| ^2 - {\mathbf {b}} : {\mathbf {q}}\right] , \end{aligned}$$

where ${\mathbf {E}}_{f({\mathbf {x}})} = \left\{ {\mathbf {q}}\left( {\mathbf {x}}\right) \in {\mathbb {R}}^{2\times 2}\,,\, q_{11}\left( {\mathbf {x}}\right) q_{22} \left( {\mathbf {x}}\right) - q_{12}\left( {\mathbf {x}}\right) q_{21}\left( {\mathbf {x}}\right) = f\left( {\mathbf {x}}\right) \right\}$, and ${\mathbf {b}}=\nabla {\mathbf {u}}^{n-1}\left( {\mathbf {x}}\right)$. Following [29, 31], we reduce the dimension of the problem with a proper change of variables. Let us introduce the vectors ${\mathbf {b}}=\left( b_{11},b_{22},b_{12},b_{21} \right)$, and ${{\mathbf {q}}}=\left( q_{11},q_{22},q_{12},q_{21} \right)$ and the $4\times 4$ matrix

$$\begin{aligned} {\mathbf {S}}= \left( \begin{array}{cccc} 1/\sqrt{2} &{} 1/\sqrt{2} &{} 0 &{} 0 \\ 1/\sqrt{2} &{} -1/\sqrt{2} &{} 0 &{} 0 \\ 0 &{} 0 &{} 1/\sqrt{2} &{} 1/\sqrt{2} \\ 0 &{} 0 &{} 1/\sqrt{2} &{} -1/\sqrt{2} \\ \end{array} \right) . \end{aligned}$$

By introducing the new variables ${\mathbf {y}}= {\mathbf {S}}{{\mathbf {q}}}^T$ and ${\mathbf {a}} = {\mathbf {S}}{{\mathbf {b}}}^T$, (16) is equivalent to

17 $$\begin{aligned} \min _{{\mathbf {y}}\in {\mathbf {F}}_{f({\mathbf {x}})}} \left[ \frac{1}{2} \left| {\mathbf {y}}\right| ^2 - {\mathbf {a}} \cdot {\mathbf {y}}\right] , \end{aligned}$$

with ${\mathbf {F}}_{f({\mathbf {x}})} = \left\{ {\mathbf {y}}\in {\mathbb {R}}^{4}\,,\, y_1^2 - y_2^2 - y_3^2 + y_4^2 = 2f({\mathbf {x}}) \right\}$. In order to solve (17), we introduce the Lagrangian functional $L$ defined by:

18 $$\begin{aligned} L \left( {\mathbf {y}}, \mu \right) = \frac{1}{2} \left| {\mathbf {y}}\right| ^2 - {\mathbf {a}} \cdot {\mathbf {y}}- \frac{\mu }{2} \left( y_1^2 - y_2^2 - y_3^2 + y_4^2 - 2f({\mathbf {x}})\right) . \end{aligned}$$

Let ${\mathbf {z}}$ denote the solution of (17), with $\lambda$ the corresponding Lagrange multiplier. The first order optimality conditions read:

19 $$\begin{aligned} \begin{aligned} z_1 - a_1&= \lambda z_1 ,\\ z_2 - a_2&= - \lambda z_2 , \\ z_3 - a_3&= - \lambda z_3 , \\ z_4 - a_4&= \lambda z_4 , \\ z_1^2 - z_2^2 - z_3^2 + z_4^2&= 2f({\mathbf {x}}). \end{aligned} \end{aligned}$$

After elimination, (19) can be solved by a (scalar) Newton method to find $\lambda$ first, and then the primal variables. Moreover, we target a solution $\lambda$ that is close to zero. Indeed, if $\lambda =0$, then $z_i = a_i$, $i=1,\ldots , 4$, and the last equation of (19) reads as

$$\begin{aligned} \frac{1}{2}\left( \det \nabla {\mathbf {u}}^{n-1}\left( {\mathbf {x}}\right) -f\left( {\mathbf {x}}\right) \right) = 0\ \ \forall {\mathbf {x}}\in \Omega , \end{aligned}$$

and therefore ${\mathbf {u}}^{n-1}$ is the solution of (1) and ${\mathbf {q}}^{n-1}=\nabla {\mathbf {u}}^{n-1}$. Numerical experiments will show that, in practise, the solution for $\lambda$ is close to zero.

When considering the Jacobian inequality (4), the solution of the minimization problem, for all ${\mathbf {x}}\in \Omega$, reads as

20 $$\begin{aligned} {\mathbf {p}}^n\left( {\mathbf {x}}\right) = \arg \min _{{\mathbf {q}}\in {\tilde{{\mathbf {E}}}}_{f(x)}} \left[ \frac{1}{2} \left| {\mathbf {q}}\right| ^2 - {\mathbf {b}} : {\mathbf {q}}\right] , \end{aligned}$$

where ${\tilde{{\mathbf {E}}}}_{f(x)} = \left\{ {\mathbf {q}}\left( {\mathbf {x}}\right) \in {\mathbb {R}}^{2\times 2}\,,\, q_{11}\left( {\mathbf {x}}\right) q_{22}\left( {\mathbf {x}}\right) - q_{12}\left( {\mathbf {x}}\right) q_{21}\left( {\mathbf {x}}\right) \ge f\left( {\mathbf {x}}\right) \right\}$ and ${\mathbf {b}}=\nabla {\mathbf {u}}^{n-1}({\mathbf {x}})$. In order to solve (20), we can introduce a slack variable and re-write the problem as

21 $$\begin{aligned} \begin{aligned} \min _{{\mathbf {q}}\in {\mathbb {R}}^{2\times 2} } \quad&\left[ \frac{1}{2} \left| {\mathbf {q}}\right| ^2 - {\mathbf {b}} : {\mathbf {q}}\right] \\ \text {s.t.} \quad&\det {\mathbf {q}}-f({\mathbf {x}})-s=0,\\&s\ge 0 . \\ \end{aligned} \end{aligned}$$

Then, a logarithmic barrier function allows to eliminate the inequality constraint (see e.g. [33]):

22 $$\begin{aligned} \begin{aligned} \min _{{\mathbf {q}}\in {\tilde{{\mathbf {E}}}}_f} \quad&\left[ \frac{1}{2} \left| {\mathbf {q}}\right| ^2 - {\mathbf {b}} : {\mathbf {q}}\right] -\mu \log s \\ \text {s.t.} \quad&\det {\mathbf {q}}-f({\mathbf {x}})-s=0,\\ \end{aligned} \end{aligned}$$

where $\mu \ge 0$. The minimization problem (22) is similar to (16). Its solution will be implemented with the same approach, relying on the same change of variables, the Lagrangian functional, and its first order optimality conditions, which are solved with a Newton method.

### Numerical Solution of the Linear Variational Problems

We focus here on the solution of (13), which is equivalent to solving:

23 $$\begin{aligned} \min _{{\mathbf {v}}\in {\bar{{\mathbf {V}}}}_g } \left[ \frac{1}{2}\int _\Omega \left| \nabla {\mathbf {v}}- {\mathbf {p}}^n \right| ^2 \text {d} {\mathbf {x}}+ \frac{\varepsilon }{2} \int _\Omega \left| \nabla ^2 {\mathbf {v}}\right| ^2dx \right] . \end{aligned}$$

We derive the first optimality conditions and obtain a fourth order partial differential equation: find ${\mathbf {u}}^{n+1/2} \in {\bar{{\mathbf {V}}}}_g$ such that

24 $$\begin{aligned} \varepsilon \int _{\Omega } \nabla ^2{\mathbf {u}}^{n+1/2} \cdot \nabla ^2 {\mathbf {v}}d{\mathbf {x}}+ \int _{\Omega } \nabla {\mathbf {u}}^{n+1/2} : \nabla {\mathbf {v}}d{\mathbf {x}}= \int _\Omega {\mathbf {p}}^n : \nabla {\mathbf {v}}d {\mathbf {x}}, \end{aligned}$$

for all $${\mathbf {v}}\in {\bar{{\mathbf {V}}}}_0 = \left\{ {\mathbf {v}}\in \left( H^1_0\left( \Omega \right) \cap H^2 \left( \Omega \right) \right) ^2 \right\} $$.

## Finite Element Approximation

Let $h>0$ be a space discretization step and let $\left\{ {\mathcal {T}}_h\right\} _h$ be family of conformal triangulations of $\Omega$ (see [38,  Appendix 1]). Let ${\mathbf {Q}}_h$ be defined as

$$\begin{aligned} {\mathbf {Q}}_h=\left\{ {\mathbf {q}}\in L^2\left( \Omega \right) ^{2\times 2}, \, {\mathbf {q}}|_{T} \in {\mathbb {R}}^{2\times 2},\,\, \forall T \in {\mathcal {T}}_h \right\} , \end{aligned}$$

equipped with the discrete inner product and corresponding norm:

$$\begin{aligned} \left( \left( {\mathbf {p}}, {\mathbf {q}}\right) \right) _{0h} = \sum _{T \in {\mathcal {T}}_h} \left| T\right| \left. {\mathbf {p}}\right| _T : \left. {\mathbf {q}}\right| _T , \qquad \left| \left| \left| {\mathbf {q}} \right| \right| \right| _{0h} = \sqrt{ \left( \left( {\mathbf {q}}, {\mathbf {q}}\right) \right) _{0h} } . \end{aligned}$$

Let ${\mathbf {Q}}_{fh}\text { and } {\tilde{{\mathbf {Q}}}}_{fh}$ be the finite dimensional subsets approximating ${\mathbf {Q}}_f$ and ${\tilde{{\mathbf {Q}}}}_f$, respectively defined by

$$\begin{aligned} {\mathbf {Q}}_{fh}= & {} \left\{ {\mathbf {q}}\in {\mathbf {Q}}_h, \,\,{\det {\mathbf {q}}}|_{T} = {\bar{f}}_T \,,\, \forall T \in {\mathcal {T}}_h \right\} , \\ {\tilde{{\mathbf {Q}}}}_{fh}= & {} \left\{ {\mathbf {q}}\in {\mathbf {Q}}_h, \,\,{\det {\mathbf {q}}}|_{T} \ge {\bar{f}}_T \,,\, \forall T \in {\mathcal {T}}_h \right\} , \end{aligned}$$

where ${\bar{f}}_T=\displaystyle \frac{1}{\vert T\vert } \int _T f\left( {\mathbf {x}}\right) d{\mathbf {x}}$. Let ${\mathbf {V}}_{{\mathbf {g}}h}\text { and }{\mathbf {V}}_{0h}$ be the finite dimensional subspaces of ${\mathbf {V}}_{\mathbf {g}}\text { and } {\mathbf {V}}_0$ given by

$$\begin{aligned} {\mathbf {V}}_{{\mathbf {g}}h}= & {} \left\{ {\mathbf {v}}\in \left( C^0\left( {\overline{\Omega }}\right) \right) ^2\,,\, {{\mathbf {v}}}|_{T} \in \left( {\mathbb {P}}_1\right) ^2, \,\, \forall T \in {\mathcal {T}}_h , \,\, {{\mathbf {v}}}|_{\Gamma _h} = {\mathbf {g}}_h \right\} , \\ {\mathbf {V}}_{0h}= & {} \left\{ {\mathbf {v}}\in \left( C^0\left( {\overline{\Omega }}\right) \right) ^2\,,\, {{\mathbf {v}}}|_{T} \in \left( {\mathbb {P}}_1\right) ^2, \,\, \forall T \in {\mathcal {T}}_h , \,\, {{\mathbf {v}}}|_{\Gamma _h} = {\mathbf {0}} \right\} , \end{aligned}$$

where ${\mathbb {P}}_1$ the space of the two-variable polynomials of degree $\le 1$, and ${\mathbf {g}}_h$ is a piecewise linear interpolant of ${\mathbf {g}}$. We define a discrete inner product and corresponding norm for ${\mathbf {V}}_{{\mathbf {g}}h}$ and ${\mathbf {V}}_{0h}$ as

$$\begin{aligned} \left( {\mathbf {u}}, {\mathbf {v}}\right) _{0h} = \sum _{T \in {\mathcal {T}}_h} \sum _{i=1}^m W_i {\mathbf {u}}\left( \zeta _i\right) \cdot {\mathbf {v}}\left( \zeta _i\right) , \qquad \left| \left| {\mathbf {u}} \right| \right| _{0h} = \sqrt{ \left( {\mathbf {u}}, {\mathbf {u}}\right) _{0h} }\ , \end{aligned}$$

with $W_i$ the weights and $\zeta _i$ the evaluation points of a Gauss quadrature rule, *m* denoting the number of points of the quadrature rule.

The discrete formulation of the least-squares method to solve (9) reads as follows: find $\mathbf {\left\{ {\mathbf {u}},{\mathbf {p}}\right\} }\in {\mathbf {V}}_{{\mathbf {g}}h} \times \ {\mathbf {Q}}_{fh}$ such that

$$\begin{aligned} J_\varepsilon \left( {\mathbf {u}},{\mathbf {p}}\right) \le J_\varepsilon \left( {\mathbf {v}},{\mathbf {q}}\right) ,\quad \forall \left\{ {\mathbf {v}},{\mathbf {q}}\right\} \in {\mathbf {V}}_{{\mathbf {g}}h} \times \ {\mathbf {Q}}_{fh}. \end{aligned}$$

### Remark 4

(*Jacobian problem with inequalities*) Similarly, the discrete formulation of the least-squares method to solve (10) reads as follows: find $\mathbf {\left\{ {\mathbf {u}},{\mathbf {p}}\right\} }\in {\mathbf {V}}_{{\mathbf {g}}h} \times \ {\tilde{{\mathbf {Q}}}}_{fh}$ such that

$$\begin{aligned} J_\varepsilon \left( {\mathbf {u}},{\mathbf {p}}\right) \le J_\varepsilon \left( {\mathbf {v}},{\mathbf {q}}\right) ,\quad \forall \left\{ {\mathbf {v}},{\mathbf {q}}\right\} \in {\mathbf {V}}_{{\mathbf {g}}h} \times \ {\mathbf {Q}}_{fh}. \end{aligned}$$

$$\square $$

The discrete formulation of the relaxation algorithm (11)–(14) described in Sect. 3.2 becomes the following:

1. We initialize the algorithm by finding ${\mathbf {u}}_h^1\in {\mathbf {V}}_{{\mathbf {g}}h}$ such that:
  25 $$\begin{aligned} \left( \left( \nabla {\mathbf {u}}_h^{1} , \nabla {\mathbf {v}}_h \right) \right) _{0h}=\left( \tilde{{\mathbf {f}}} , {\mathbf {v}}_h \right) , \ \forall {\mathbf {v}}_h\in {\mathbf {V}}_{0h}, \end{aligned}$$
  where $\tilde{{\mathbf {f}}}=\left( 1,1\right) ^T$ for all ${\mathbf {x}}\in \Omega$. Then, for $n\ge 1$,
2. We solve the discrete local nonlinear problem:
  26 $$\begin{aligned} {\mathbf {p}}^n_h = \arg \min _{{\mathbf {q}}\in {\mathbf {Q}}_{fh} } \left[ \frac{1}{2} \left( \left( {\mathbf {q}}, {\mathbf {q}}\right) \right) _{0h}^2 - \left( \left( \nabla {\mathbf {u}}_h^{n} , {\mathbf {q}}\right) \right) _{0h} \right] . \end{aligned}$$
  When considering the Jacobian problem with inequality constraints (4), we replace ${\mathbf {Q}}_{fh}$ by ${\tilde{{\mathbf {Q}}}}_{fh}$ in (26). The solution of the discrete minimization problem (26), ${\mathbf {p}}_h^n$, is obtained on each element *T* of ${\mathcal {T}}_h$, in an identical manner as the solution of the continuous problem described in Sect. 3.3.
3. We solve the discrete linear variational problem:
  $$\begin{aligned} {\mathbf {u}}_h^{n+1/2} = \arg \min _{{\mathbf {v}}\in {\mathbf {V}}_{{\mathbf {g}}h}} J_\varepsilon \left( {\mathbf {v}},{\mathbf {p}}_h^n\right) . \end{aligned}$$
  The first order optimality conditions read: find ${\mathbf {u}}_h^{n+1/2} \in {\mathbf {V}}_{{\mathbf {g}}h}$ satisfying
  27 $$\begin{aligned} \varepsilon \left( \nabla ^2{\mathbf {u}}_h^{n+1/2}, \nabla ^2 {\mathbf {v}}_h \right) _{0h} + \left( \left( \nabla {\mathbf {u}}_h^{n+1/2} , \nabla {\mathbf {v}}_h \right) \right) _{0h} = \left( \left( {\mathbf {p}}^n_h , \nabla {\mathbf {v}}_h \right) \right) _{0h}, \end{aligned}$$
  for all ${\mathbf {v}}_h \in {\mathbf {V}}_{0h}$. By applying a mixed finite element method to (27), we solve an equivalent augmented system of equations: find $\left\{ {\mathbf {u}}_h^{n+1/2}, {\mathbf {w}}_h^n\right\} \in {\mathbf {V}}_{{\mathbf {g}}h}\times {\mathbf {V}}_{0h}$ such that,
  $$\begin{aligned} \varepsilon \left( \left( \nabla {\mathbf {w}}_h^n ,\nabla {\mathbf {v}}_h \right) \right) _{0h} + \left( {\mathbf {w}}_h^n ,{\mathbf {v}}_h\right) _{0h}= & {} \left( \left( {\mathbf {p}}^n_h, \nabla {\mathbf {v}}_h \right) \right) _{0h}, \\ \left( \left( \nabla {\mathbf {u}}_h^{n+1/2} , \nabla \varvec{\varphi }_h \right) \right) _{0h}= & {} \left( {\mathbf {w}}_h^n,\varvec{\varphi }_h\right) _{0h}, \end{aligned}$$
  for all $$\left\{ {\mathbf {v}}_h,\varvec{\varphi }_h \right\} \in {\mathbf {V}}_{0h} \times {\mathbf {V}}_{0h}$$.
4. We update the solution by:
  $$\begin{aligned} {\mathbf {u}}^{n+1}_h = {\mathbf {u}}^{n}_h + \omega \left( {\mathbf {u}}^{n+1/2}_h - {\mathbf {u}}^n_h\right) . \end{aligned}$$

## Numerical Experiments

We validate the convergence and accuracy of the proposed least-squares method with various numerical experiments. We consider several domains, namely the unit square $\Omega _q=\left( 0,1\right) ^2$, the unit disk $\Omega _d=\left\{ {\mathbf {x}}\in {\mathbb {R}}^2,\vert \vert {\mathbf {x}}\vert \vert _2 <1 \right\}$, the so-called ’pacman’ domain

$$\begin{aligned} \Omega _p=\Omega _d{\setminus } \left\{ (x_1,x_2)\in {\mathbb {R}}^2, x>0, \left| x_2\right| <x_1\right\} , \end{aligned}$$

and the cracked unit disk

$$\begin{aligned} \Omega _c=\Omega _d{\setminus } \left\{ (x_1,x_2)\in {\mathbb {R}}^2, x>0, \left| x_2\right| <\tan \left( \frac{\pi }{100}\right) x_1\right\} . \end{aligned}$$

**Fig. 1:**
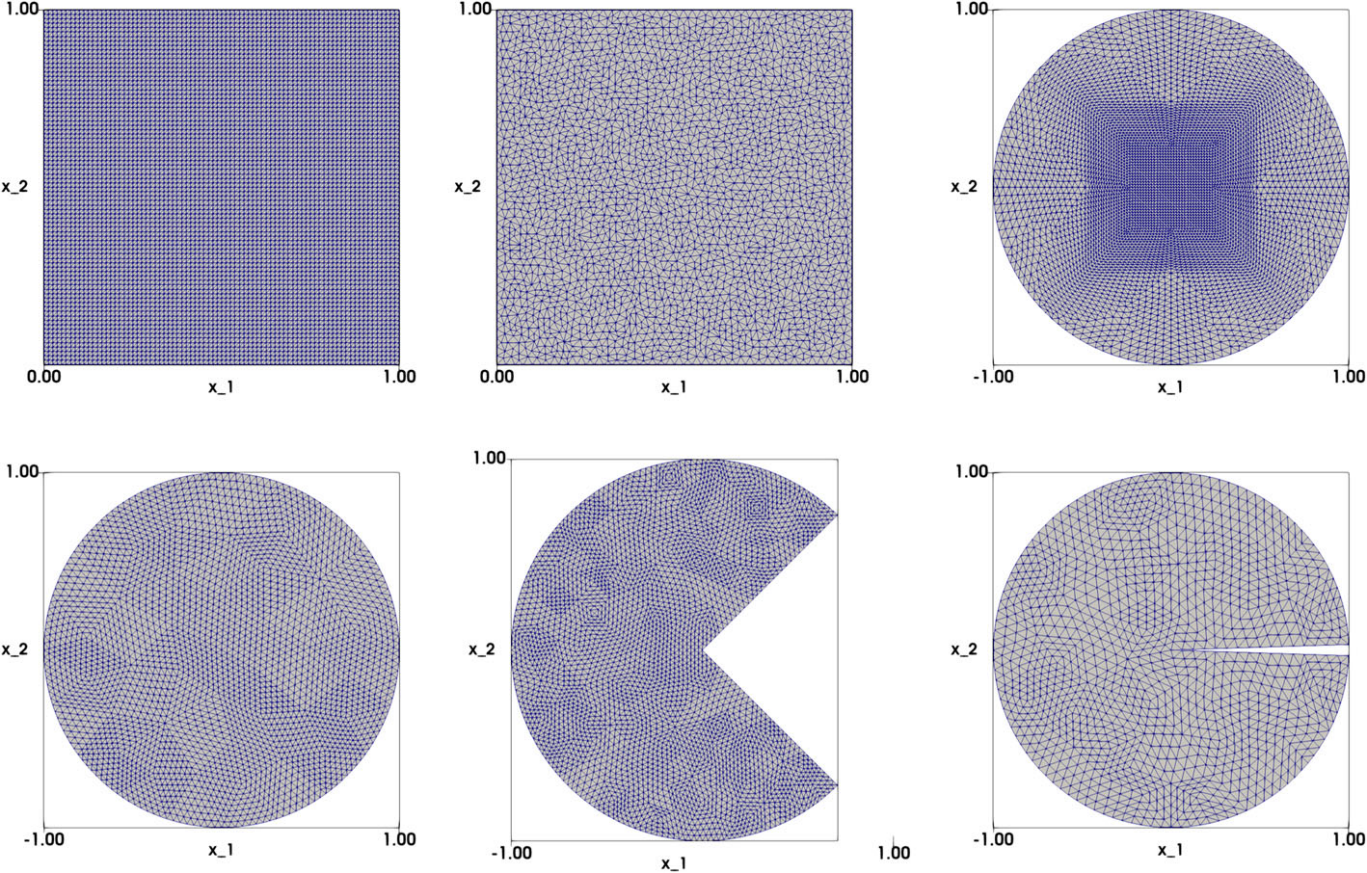
Finite element meshes used for the numerical experiments. Top left: structured mesh for the unit square ($\Omega _q = (0,1)^2$, $h=0.0125$); Top middle: unstructured mesh for the unit square ($\Omega _q = (0,1)^2$, $h=0.01882$); Top right: structured mesh for the unit disk ($\Omega _d = \{(x_1,x_2) \in {\mathbb {R}}^2 : x_1^2+x_2^2<1\}$, $h\simeq 0.0209$); Bottom left: unstructured mesh for the unit disk ($\Omega _d = \{(x_1,x_2) \in {\mathbb {R}}^2 : x_1^2+x_2^2<1\}$, $h\simeq 0.08$); Bottom middle: unstructured mesh for the pacman domain ($\Omega _p =\Omega _d{\setminus } \left\{ (x_1,x_2)\in {\mathbb {R}}^2, x_1>0, \left| x_2\right| <x_1\right\}$, $h=0.0252$); Bottom right: unstructured mesh for the cracked unit disk ($\Omega _c = \Omega _d{\setminus } \left\{ (x_1,x_2)\in {\mathbb {R}}^2, x_1>0, \left| x_2\right| <\tan \left( \frac{\pi }{100}\right) x_1\right\}$, $h=0.0486$)

Figure 1 illustrates typical triangulations of these domains. For coarse meshes, we initialize the algorithm by solving (25) with given boundary data. For finer meshes, we initialize the algorithm by using the numerical solution obtained on a coarser mesh. In all experiments, $\varepsilon \simeq h^2$ (unless specified otherwise). The relaxation parameter is $\omega \simeq 1$ initially, and then gradually increases to $\omega = 2$. The stopping criterion for the relaxation algorithm is $\vert \vert {\mathbf {u}}_h^n-{\mathbf {u}}_h^{n-1} \vert \vert _{0h}< 10^{-8}$. The Newton’s method to solve the local nonlinear problems stops when the difference between two successive iterations is smaller than $10^{-15}$. Usually, this number of iterations does not exceed 5. The interior-point parameter $\mu$ is specified later.

The estimation of $\lambda$ is denoted by ${\bar{\lambda }}$, and is obtained by averaging its values $\lambda _T$ on each triangle, for each triangle $T\in {\mathcal {T}}_h$. It is accompanied by the standard deviation of the estimator, ${\bar{\sigma }}$, to describe the variability over the mesh elements. These indicators are:

$$\begin{aligned} {\bar{\lambda }}=\frac{\displaystyle \sum _{T \in {\mathcal {T}}_h} \lambda _T}{N}, \qquad {\bar{\sigma }}=\sqrt{\frac{ \displaystyle \sum _{T \in {\mathcal {T}}_h} \left( \lambda _T-{\bar{\lambda }}\right) ^2}{N-1}}, \end{aligned}$$

where *N* is the number of triangles of the triangulation ${\mathcal {T}}_h$.

### Numerical Approximation of the Identity Map

The first experiment corresponds to considering the identity map as the exact solution. The data are chosen as $f=1$ and ${\mathbf {u}}\left( {\mathbf {x}}\right) ={\mathbf {x}}$ on $\Gamma$. The problem can be written as: find ${\mathbf {u}}: \Omega \rightarrow {\mathbb {R}}^2$ such that:

28 $$\begin{aligned} \left\{ \begin{array}{ll} \det {\nabla {\mathbf {u}}\left( {\mathbf {x}}\right) }=1 &{}\text {in} \ \Omega ,\\ \\ {\mathbf {u}}\left( {\mathbf {x}}\right) ={\mathbf {x}}&{} \text {on} \ \Gamma . \end{array}\right. \end{aligned}$$

**Fig. 2:**
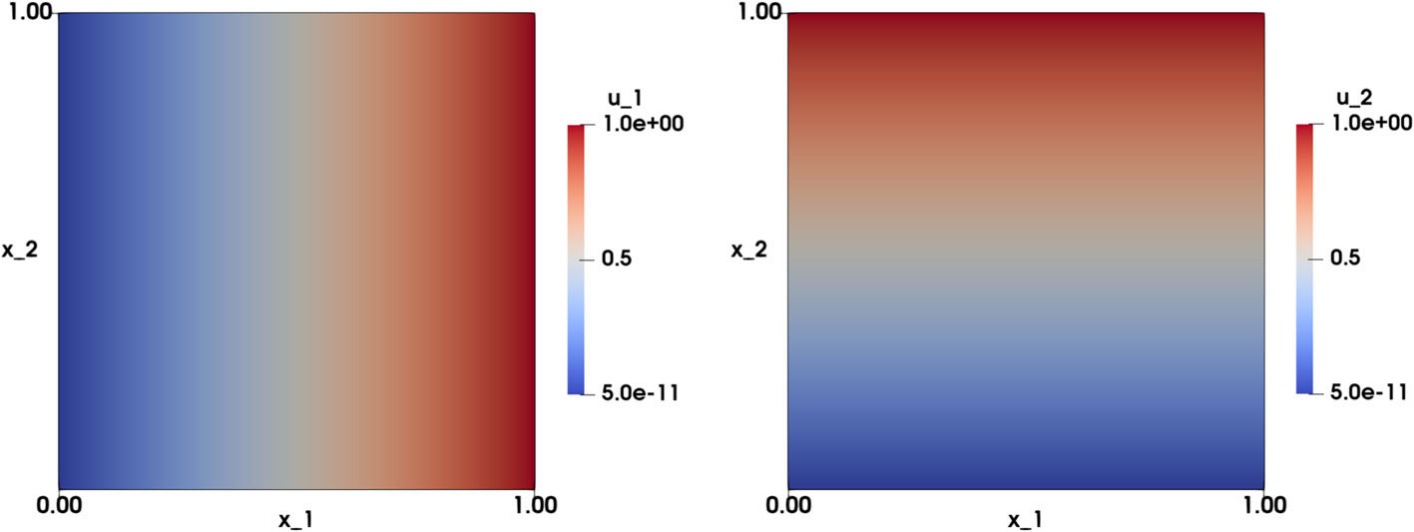
Identity map test case on the unit square with data $f=1$ and ${\mathbf {g}}({\mathbf {x}})={\mathbf {x}}$. Visualization of the numerical approximation of the solution ${\mathbf {u}}_h$. Left: the component $u_{1,h}$; Right: the component $u_{2,h}$. Results are obtained on a structured mesh with $h=0.0125$

When $\Omega = \Omega _q =\left( 0,1 \right) ^2$, we use structured meshes with mesh size $h=\left\{ 0.00625, 0.025,\right. \left. 0.0125,0.05\right\}$, The errors for the obtained approximations are of order $10^{-10}$ in the $L^2\left( \Omega \right)$ error norm, and of order $10^{-9}$ to $10^{-10}$ in the $H^1\left( \Omega \right)$ error norm. In addition, $\vert \vert \nabla {\mathbf {u}}_h-{\mathbf {p}}_h\vert \vert _{0h}$ and ${\bar{\lambda }}$ are of the order of $10^{-10}$. Figure 2 illustrates the approximation on the structured mesh of the two components of the numerical solution. More precisely, with $h=0.025$, after 29 iterations, we obtain

$$\begin{aligned} \vert \vert {\mathbf {u}}-{\mathbf {u}}_h\vert \vert _{L^2\left( \Omega \right) }=1.24\cdot 10^{-10}, \quad \vert {\mathbf {u}}-{\mathbf {u}}_h\vert _{H^1\left( \Omega \right) }=7.09\cdot 10^{-10}, \end{aligned}$$

and

$$\begin{aligned} \vert \vert \nabla {\mathbf {u}}_h - {\mathbf {p}}_h\vert \vert _{0h}=4.21\cdot 10^{-10} , \quad {\bar{\lambda }}=2.96\cdot 10^{-10}. \end{aligned}$$

When $\Omega =\Omega _d = \{\left( x_1,x_2\right) \in {\mathbb {R}}^2, x_1^2+x_2^2<1\}$, we use structured meshes with mesh size $h=\left\{ 0.0831, 0.0421,0.0209, 0.0104\right\}$. Similar to when working on the unit square, the errors for the approximations are again of order $10^{-10}$ in the $L^2\left( \Omega \right)$ error norm, and of order $10^{-9}$ to $10^{-10}$ in the $H^1\left( \Omega \right)$ error norm. Furthermore, $\vert \vert \nabla {\mathbf {u}}_h-{\mathbf {p}}_h\vert \vert _{0h}$ and ${\bar{\lambda }}$ are of the order of $10^{-10}$ to $10^{-11}$. In particular, for $h=0.025$, and after 29 iterations, we obtain

$$\begin{aligned} \vert \vert {\mathbf {u}}-{\mathbf {u}}_h\vert \vert _{L^2\left( \Omega \right) }=2.06\cdot 10^{-10} , \quad \vert {\mathbf {u}}-{\mathbf {u}}_h\vert _{H^1\left( \Omega \right) }=7.24\cdot 10^{-10}, \end{aligned}$$

and

$$\begin{aligned} \vert \vert \nabla {\mathbf {u}}_h-{\mathbf {p}}_h\vert \vert _{0h}=2.06\cdot 10^{-10} , \quad {\bar{\lambda }}=9.77\cdot 10^{-11}. \end{aligned}$$

**Fig. 3:**
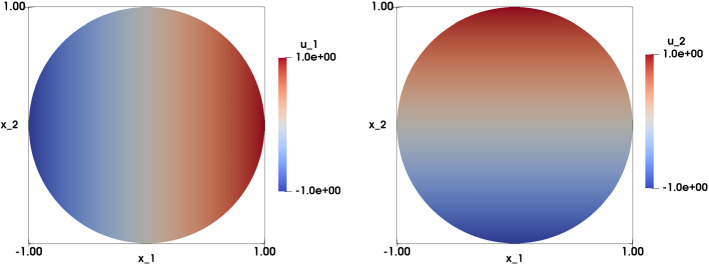
Identity map test case on the unit disk with data $f=1$ and ${\mathbf {g}}({\mathbf {x}})={\mathbf {x}}$. Visualization of the numerical approximation of the solution ${\mathbf {u}}_h$. Left: the component $u_{1,h}$; Right: the component $u_{2,h}$. Results are obtained on a structured mesh with $h=0.0209$

Figure 3 illustrates the approximation of the two components of the numerical solution on the structured mesh of the unit disk.

Then, the algorithm is tested by considering the inequality problem (4). We consider the following problem ($f=0$, ${\mathbf {g}}({\mathbf {x}})={\mathbf {x}}$ and $\Omega =\Omega _d = \{\left( x_1,x_2\right) \in {\mathbb {R}}^2, x_1^2+x_2^2<1\}$):

29 $$\begin{aligned} \left\{ \begin{array}{ll} \det {\nabla {\mathbf {u}}\left( {\mathbf {x}}\right) }\ge 0 &{} \text {in} \ \Omega ,\\ \\ {\mathbf {u}}\left( {\mathbf {x}}\right) ={\mathbf {x}}&{} \text {on} \ \Gamma . \end{array}\right. \end{aligned}$$

There is some information missing in (29) since $f=0$, and the only available relevant information on the solution comes from the boundary conditions. The algorithm converges to an exact solution ${\mathbf {u}}\left( {\mathbf {x}}\right) ={\mathbf {x}}$, in a similar fashion as for (28). Actually the solution obtained when solving (29) is similar to the one obtained by solving (28).

In this test case we choose $\mu =0.1$ and we decrease it with a factor of $\sqrt{h}$ at each iteration. By contrast to solving the Jacobian equality in (28), numerical errors in $L^2\left( \Omega \right)$ and $H^1\left( \Omega \right)$ norms of solving the inequality (29) using the same mesh size values are of the order of $10^{-2}$ and $10^{-3}$, respectively. These parameters depend on the choice of $\mu$: if initially $\mu$ is chosen to be large, $\vert \vert \nabla {\mathbf {u}}_h-{\mathbf {p}}_h\vert \vert _{0h}$ will be also large; if $\mu$ is small, we experience some convergence problems. When considering $h=0.025$, we obtain after 69 iterations:

$$\begin{aligned} \vert \vert {\mathbf {u}}-{\mathbf {u}}_h\vert \vert _{L^2\left( \Omega \right) }=6.86\cdot 10^{-10} , \quad \vert {\mathbf {u}}-{\mathbf {u}}_h\vert _{H^1\left( \Omega \right) }=2.67\cdot 10^{-9} , \end{aligned}$$

and

$$\begin{aligned} \vert \vert \nabla {\mathbf {u}}_h-{\mathbf {p}}_h\vert \vert _{0h}= 1.04\cdot 10^{-2} , \quad {\bar{\lambda }}= 2.08\cdot 10^{-3}. \end{aligned}$$

### Smooth Solution with Radial Right-Hand Side

Let $\Omega =\{(x_1,x_2)\in {\mathbb {R}}^2, x_1^2+x_2^2<1\}$ be the unit disk. We consider the following problem: find ${\mathbf {u}}: \Omega \rightarrow {\mathbb {R}}^2$ such that:

30 $$\begin{aligned} \left\{ \begin{array}{ll} \det {\nabla {\mathbf {u}}}({\mathbf {x}}) = f({\mathbf {x}}) &{} \text {in} \ \Omega ,\\ \\ {\mathbf {u}}\left( {\mathbf {x}}\right) ={\mathbf {g}}\left( {\mathbf {x}}\right) &{} \text {on} \ \Gamma , \end{array}\right. \end{aligned}$$

where

31 $$\begin{aligned} f({\mathbf {x}}) = 2\left( x_1^2+x_2^2\right) , \quad {\mathbf {g}}\left( {\mathbf {x}}\right) =\sqrt{2} \begin{pmatrix} \frac{1}{2}\left( x_1^2-x_2^2\right) \\ x_1 x_2 \end{pmatrix}. \end{aligned}$$

One exact solution satisfies

32 $$\begin{aligned} {\mathbf {u}}\left( {\mathbf {x}}\right) =\sqrt{2} \begin{pmatrix} \frac{1}{2}\left( x_1^2-x_2^2\right) \\ x_1 x_2 \end{pmatrix}. \end{aligned}$$

**Fig. 4:**
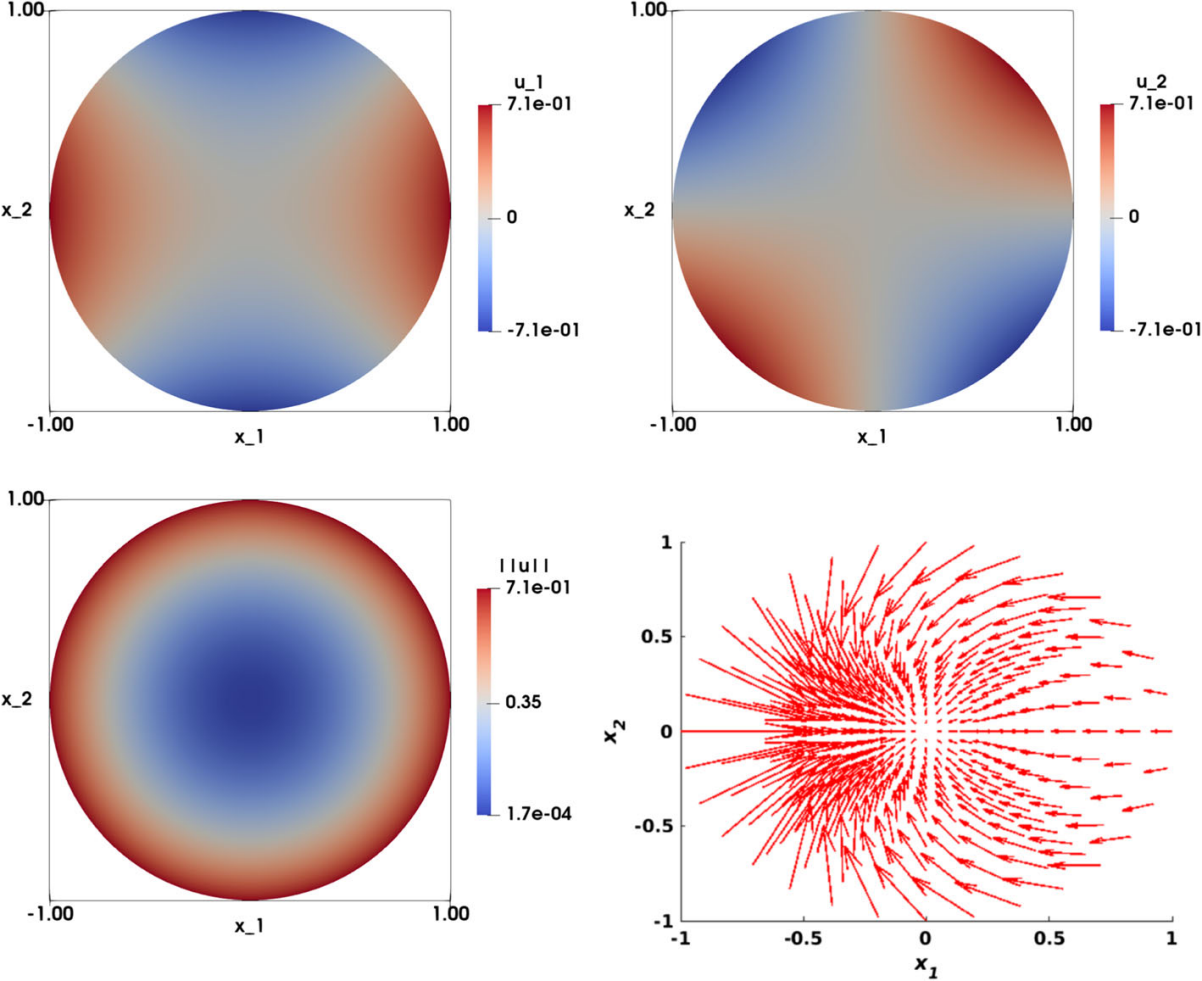
Smooth solution with radial right-hand side test case ($f(x_1,x_2)=2\left( x_1^2+x_2^2\right)$ and ${\mathbf {g}}(x_1,x_2)=\sqrt{2}\left( \frac{1}{2}\left( x_1^2-x_2^2\right) ,x_1x_2 \right) ^T$ on $\Gamma$). Top left: Numerical approximation of the solution of the component $u_{1,h}$. Top right: Numerical approximation of the solution of the component $u_{2,h}$. Bottom left: Visualization of $\left| \left| {\mathbf {u}}_h\right| \right|$. Bottom right: Visualization of the vector field ${\mathbf {u}}_h$. The results are obtained on structured mesh of the unit disk with $h=0.0209$

The non-uniqueness of the solution of Eq. (30) makes this test case challenging. For instance, this may cause the algorithm to fluctuate between two different solutions. Later on, we show that our algorithm converges to a solution for different sets of parameters. The numerical solution of (30) is illustrated in Fig. 4 (top row). Note that both the right-hand side of this equation and $\left| \left| {\mathbf {u}}_h\right| \right|$ (bottom left), are radial symmetric. However, the solution ${\mathbf {u}}_h$ does not exhibit the same symmetry pattern.

Table 1 provides insights about the convergence of the relaxation algorithm towards the numerical solution on the structured mesh for the unit disk. The only difference with the results presented in the previous section is that the numerical solution converges in $L^2$-norm with a nearly optimal rate of $O\left( h^{1.7}\right)$ to $O\left( h^{2}\right)$, and in $H^1$ semi-norm with an optimal rate of $O\left( h\right)$. Similar results are observed in Table 2, where the unstructured mesh is used. We observe that the numerical solution converges in $L^2$-norm and $H^1$-semi norm with rates of $O\left( h^{1.9}\right)$ and *O*(*h*), respectively. This confirms that the behavior of the algorithm does not depend on the structure of the mesh.

**Table 1:** Smooth solution with radial right-hand side test case. The case of the unit disk with a structured mesh. ($f(x_1,x_2)=2\left( x_1^2+x_2^2\right)$ and ${\mathbf {g}}(x_1,x_2)=\sqrt{2}\left( \frac{1}{2}\left( x_1^2-x_2^2\right) ,x_1x_2 \right) ^T$ on $\Gamma$). Computational results include the mesh size *h*, the $L^2$ and $H^1$ error norms with the corresponding rates, the error $\left| \left| \nabla {\mathbf {u}}_h-{\mathbf {p}}_h\right| \right| _{L^2\left( \Omega \right) }$, the average value ${\bar{\lambda }}$ and its standard deviation ${\bar{\sigma }}$, and the number of iterations of the relaxation algorithm

*h*	$\vert \vert {\mathbf {u}}-{\mathbf {u}}_h\vert \vert _{L^2\left( \Omega \right) }$		$\vert {\mathbf {u}}-{\mathbf {u}}_h\vert _{H^1\left( \Omega \right) }$		$\vert \vert \nabla {\mathbf {u}}_h-{\mathbf {p}}_h\vert \vert _{L^2\left( \Omega \right) }$	${\bar{\lambda }}({\bar{\sigma }})$	iter
0.0831	5.95e$-$03		2.14e$-$01		9.05e$-$02	1.37e$-$02(0.063)	19
0.0421	1.27e$-$03	2.19	1.03e$-$01	1.05	4.25e$-$02	7.50e$-$03(0.036)	39
0.0209	3.38e$-$04	1.91	5.22e$-$02	0.98	2.17e$-$02	3.58e$-$03(0.020)	79
0.0104	1.05e$-$04	1.70	2.63e$-$02	0.98	1.10e$-$02	1.76e$-$03(0.011)	219

**Table 2:** Smooth solution with radial right-hand side test case. The case of the unit disk with an unstructured mesh. ($f(x_1,x_2)=2\left( x_1^2+x_2^2\right)$ and ${\mathbf {g}}(x_1,x_2)=\sqrt{2}\left( \frac{1}{2}\left( x_1^2-x_2^2\right) ,x_1x_2 \right) ^T$ on $\Gamma$). Computational results include the mesh size *h*, the $L^2$ and $H^1$ error norms with the corresponding rates, the error $\left| \left| \nabla {\mathbf {u}}_h-{\mathbf {p}}_h\right| \right| _{L^2\left( \Omega \right) }$, the average value ${\bar{\lambda }}$ and its standard deviation ${\bar{\sigma }}$, and the number of iterations of the relaxation algorithm

*h*	$\vert \vert {\mathbf {u}}-{\mathbf {u}}_h\vert \vert _{L^2\left( \Omega \right) }$		$\vert {\mathbf {u}}-{\mathbf {u}}_h\vert _{H^1\left( \Omega \right) }$		$\vert \vert \nabla {\mathbf {u}}_h-{\mathbf {p}}_h\vert \vert _{L^2\left( \Omega \right) }$	${\bar{\lambda }}({\bar{\sigma }})$	iter
0.1327	5.79e$-$03		2.25e$-$01		1.14e$-$01	1.31e$-$02(0.062)	19
0.0665	1.53e$-$03	1.92	1.13e$-$01	1.00	5.74e$-$02	5.88e$-$03(0.038)	29
0.0332	4.06e$-$04	1.92	5.65e$-$02	1.00	2.85e$-$02	2.15e$-$03(0.022)	69
0.0166	1.30e$-$04	1.64	2.83e$-$02	1.00	1.42e$-$02	1.02e$-$03(0.012)	199

In a second step, we consider the Jacobian inequality problem (4). Let us consider the unit disk $\Omega =\{(x_1,x_2)\in {\mathbb {R}}^2, x_1^2+x_2^2<1\}$, together with $f\equiv 0$, and the same function ${\mathbf {g}}({\mathbf {x}})$ from (31). Numerical results show that the numerical approximation of the solution to the inequality problem obtained is the same as the one of the Jacobian equality problem (32). Table 3 shows results obtained with the structured mesh. The parameter $\mu$ for the solution of the local non-linear problems is chosen to be 0.1 and we decrease it by a factor $0.001\sqrt{h}$ at each iteration of the interior point method. We can observe that the numerical solution in this case converges in $L^2$-norm and $H^1$-semi norm with a rate of $O\left( h^{1.9}\right)$ (nearly optimal) and *O*(*h*) (optimal), respectively.

**Table 3:** Smooth solution of the Jacobian inequality problem with radial right-hand side test case. The case of the unit disk with a structured mesh. ($f(x_1,x_2)=0$ and ${\mathbf {g}}(x_1,x_2)=\sqrt{2}\left( \frac{1}{2}\left( x_1^2-x_2^2\right) ,x_1x_2 \right) ^T$ on $\Gamma$). Computational results include the mesh size *h*, the $L^2$ and $H^1$ error norms with the corresponding rates, the error $\left| \left| \nabla {\mathbf {u}}_h-{\mathbf {p}}_h\right| \right| _{L^2\left( \Omega \right) }$, the average value ${\bar{\lambda }}$ and its standard deviation ${\bar{\sigma }}$, and the number of iterations of the relaxation algorithm

*h*	$\vert \vert {\mathbf {u}}-{\mathbf {u}}_h\vert \vert _{L^2\left( \Omega \right) }$		$\vert {\mathbf {u}}-{\mathbf {u}}_h\vert _{H^1\left( \Omega \right) }$		$\vert \vert \nabla {\mathbf {u}}_h-{\mathbf {p}}_h\vert \vert _{L^2\left( \Omega \right) }$	${\bar{\lambda }}({\bar{\sigma }})$	iter
0.0831	6.12e$-$03		2.14e$-$01		1.08e$-$03	7.42e$-$04(0.010)	59
0.0421	1.33e$-$03	2.20	1.03e$-$01	1.05	4.74e$-$04	2.94e$-$04(0.007)	99
0.0209	3.48e$-$04	1.93	5.23e$-$02	0.98	3.20e$-$04	2.42e$-$04(0.007)	119
0.0104	9.36e$-$05	1.89	2.63e$-$02	0.99	2.09e$-$04	1.62e$-$04(0.005)	219

### Smooth Radial Solution with Non-smooth Gradient

This numerical experiment introduces a singularity in the gradient of the solution. Let $\Omega =\{(x_1,x_2)\in {\mathbb {R}}^2, x_1^2+x_2^2<1\}$ be the unit disk, $f(x_1,x_2)=2\left( x_1^2+x_2^2\right)$, and ${\mathbf {g}}\left( {\mathbf {x}}\right) ={\mathbf {x}}$ in (1). Problem (1) therefore admits an exact solution in that case, given by:

$$\begin{aligned} {\mathbf {u}}\left( x_1,x_2\right) =\sqrt{x_1^2+x_2^2} \begin{pmatrix} x_1\\ x_2 \end{pmatrix}, \end{aligned}$$

whose gradient is

$$\begin{aligned} \nabla {\mathbf {u}}\left( x_1,x_2\right) = \frac{1}{\sqrt{x_1^2+x_2^2}} \begin{pmatrix} 2x_1^2 + x_2^2 &{} x_1x_2\\ x_1x_2 &{} x^2 + 2x_2^2 \end{pmatrix}. \end{aligned}$$

**Fig. 5:**
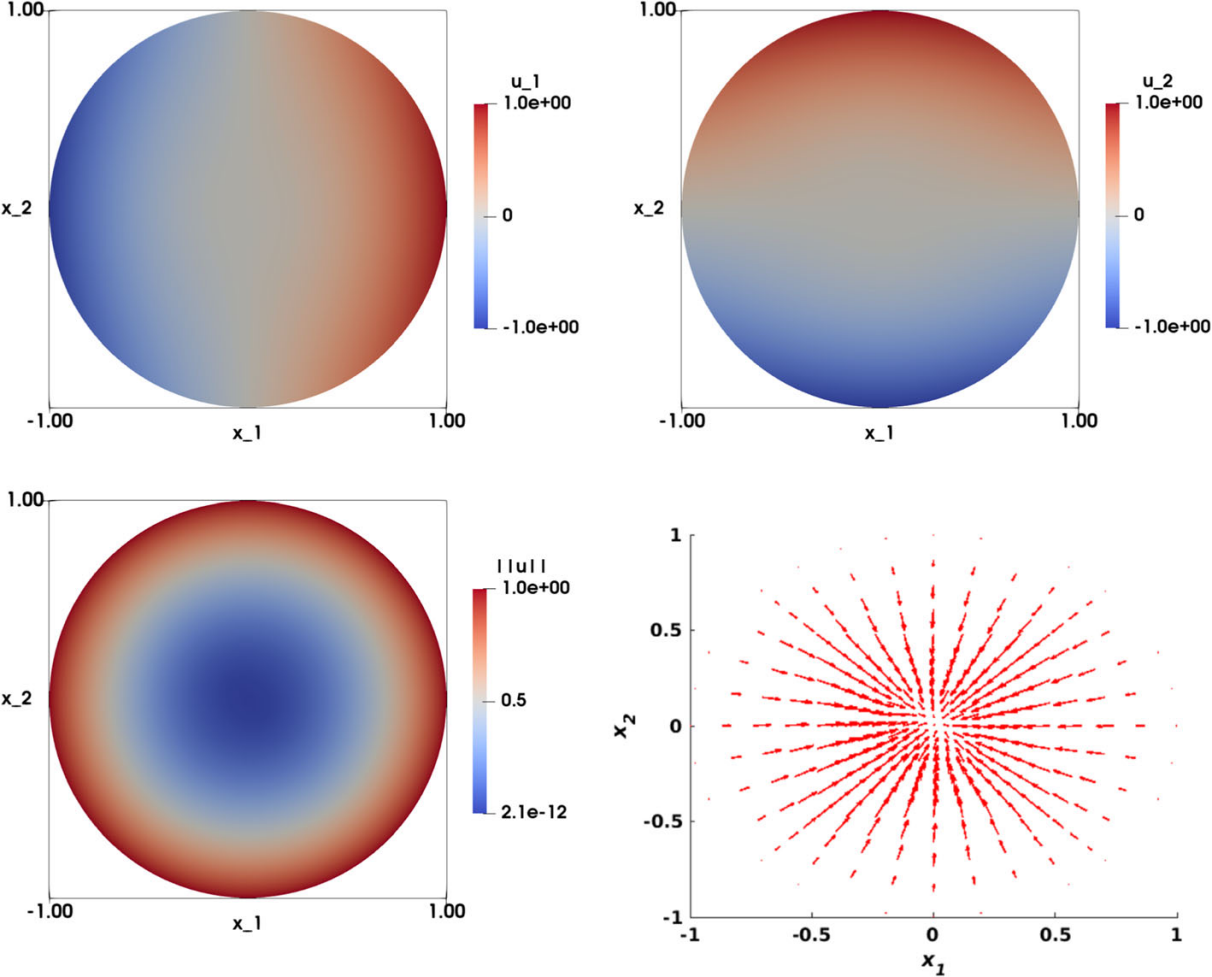
Smooth radial symmetric solution with non-smooth gradient ($f(x_1,x_2)=2\left( x_1^2+x_2^2\right)$ and ${\mathbf {g}}(x_1,x_2)=\left( x_1,x_2 \right) ^T$ on $\Gamma$). Top left: Numerical approximation of the solution of the component $u_{1,h}$. Top right: Numerical approximation of the solution of the component $u_{2,h}$. Bottom left: Visualization of $\left| \left| {\mathbf {u}}_h\right| \right|$. Bottom right: Visualization of the vector field ${\mathbf {u}}_h$. The results are obtained on structured mesh of the unit disk with $h=0.0209$

Note that the solution ${\mathbf {u}}$ is a smooth radial function, but $\nabla {\mathbf {u}}$ is not defined at the origin (0, 0). Figure 5 visualizes the numerical solution. We can notice that i) both components of the solution are smooth (first row), ii) the norm $\vert \vert {\mathbf {u}}_h\vert \vert _{0h}$ is radially symmetric (bottom left), and iii) the solution field is directed towards the origin (bottom right). Convergence properties of the relaxation algorithm on the structured mesh on the unit disk are presented in Table 4. We see that the numerical solution converges with a rate of $O\left( h^{1.1}\right)$ to $O\left( h^{1.5}\right)$, in $L^2$-norm, and with an optimal rate of $O\left( h\right)$ in the $H^1$ semi norm. This test case is more computationally expensive, and the maximum allowed number of iterations may be reached. Similar results are reported in Table 5 when using unstructured meshes.

**Table 4:** Smooth solution with radial right-hand side test case. The case of the unit disk with a structured mesh. ($f(x_1,x_2)=2\left( x_1^2+x_2^2\right)$ and ${\mathbf {g}}(x_1,x_2)=\left( x_1,x_2 \right) ^T$ on $\Gamma$). Computational results include the mesh size *h*, the $L^2$ and $H^1$ error norms with the corresponding rates, the error $\left| \left| \nabla {\mathbf {u}}_h-{\mathbf {p}}_h\right| \right| _{L^2\left( \Omega \right) }$, the average value ${\bar{\lambda }}$ and its standard deviation ${\bar{\sigma }}$, and the number of iterations of the relaxation algorithm

*h*	$\vert \vert {\mathbf {u}}-{\mathbf {u}}_h\vert \vert _{L^2\left( \Omega \right) }$		$\vert {\mathbf {u}}-{\mathbf {u}}_h\vert _{H^1\left( \Omega \right) }$		$\vert \vert \nabla {\mathbf {u}}_h-{\mathbf {p}}_h\vert \vert _{L^2\left( \Omega \right) }$	${\bar{\lambda }}({\bar{\sigma }})$	iter
0.0831	1.57e$-$01		6.44e$-$01		3.84e$-$01	$-$2.72e$-$01(0.270)	999
0.0421	6.60e$-$02	1.25	3.18e$-$01	1.02	1.73e$-$01	$-$1.13e$-$01(0.129)	199
0.0209	2.62e$-$02	1.33	1.56e$-$01	1.02	7.87e$-$02	$-$3.72e$-$02(0.057)	309
0.0104	9.20e$-$03	1.51	7.07e$-$02	1.14	3.31e$-$02	$-$1.07e$-$02(0.023)	999

**Table 5:** Smooth solution with radial right-hand side test case. The case of the unit disk with an unstructured mesh. ($f(x_1,x_2)=2\left( x_1^2+x_2^2\right)$ and ${\mathbf {g}}(x_1,x_2)=\left( x_1,x_2 \right) ^T$ on $\Gamma$). Computational results include the mesh size *h*, the $L^2$ and $H^1$ error norms with the corresponding rates, the error $\left| \left| \nabla {\mathbf {u}}_h-{\mathbf {p}}_h\right| \right| _{L^2\left( \Omega \right) }$, the average value ${\bar{\lambda }}$ and its standard deviation ${\bar{\sigma }}$, and the number of iterations of the relaxation algorithm

*h*	$\vert \vert {\mathbf {u}}-{\mathbf {u}}_h\vert \vert _{L^2\left( \Omega \right) }$		$\vert {\mathbf {u}}-{\mathbf {u}}_h\vert _{H^1\left( \Omega \right) }$		$\vert \vert \nabla {\mathbf {u}}_h-{\mathbf {p}}_h\vert \vert _{L^2\left( \Omega \right) }$	${\bar{\lambda }}({\bar{\sigma }})$	iter
0.1327	1.71e$-$01		6.77e$-$01		4.40e$-$01	$-$9.82e$-$02(0.244)	359
0.0665	7.39e$-$02	1.21	3.19e$-$01	1.09	1.93e$-$01	$-$5.24e$-$02(0.130)	259
0.0332	2.65e$-$02	1.48	1.39e$-$01	1.20	7.44e$-$02	$-$1.86e$-$02(0.059)	489
0.0166	9.51e$-$03	1.48	6.17e$-$02	1.17	2.87e$-$02	$-$5.31e$-$03(0.026)	999

### Smooth Solution with Radial Right-Hand Side on Non-convex Domains

In this experiment we revisit the case presented in Sect. 5.2 on non-convex domains $\Omega$. Generally speaking, the existence of a solution to a fully nonlinear equation in non-convex domains is not guaranteed, see, e.g., [39]. Nevertheless, we treat here one case that admits an exact solution. We focus on the following problem: find ${\mathbf {u}}: \Omega \rightarrow {\mathbb {R}}^2$ such that:

33 $$\begin{aligned} \left\{ \begin{array}{lll} \det {\nabla {\mathbf {u}}}(x_1,x_2) &{}=2(x_1^2 + x_2^2) \ &{} \text {in} \ \Omega ,\\ \\ {\mathbf {u}}\left( x_1,x_2 \right) &{}=\sqrt{2} \begin{pmatrix} \frac{1}{2}\left( x_1^2-x_2^2\right) \\ x_1 x_2 \end{pmatrix} \&\text {on} \ \Gamma . \end{array}\right. \end{aligned}$$

In this case, the exact solution in $\Omega$ is

$$\begin{aligned} {\mathbf {u}}\left( x_1,x_2\right) =\sqrt{2} \begin{pmatrix} \frac{1}{2}\left( x_1^2-x_2^2\right) \\ x_1 x_2 \end{pmatrix} \end{aligned}$$

Tables 6 and 7 illustrate the results for both non-convex domains (pacman with unstructured mesh, and cracked disk with an unstructured mesh respectively). We see that the numerical solutions converge in $L^2$-norm with a rate of $O\left( h^{1.9}\right)$ to $O\left( h^{1.7}\right)$ and $O\left( h^{1.8}\right)$, respectively. Convergence in $H^1$ semi norm is of (optimal) order *O*(*h*) for both domains. The number of iterations of the relaxation algorithm is linearly increasing for decreasing *h*. Comparing these two tables, we can say that the performance of the algorithm is the same for the two non-convex domains. Comparing with Table 1, the algorithm has the same level of performance for both convex and non-convex domains.

**Fig. 6:**
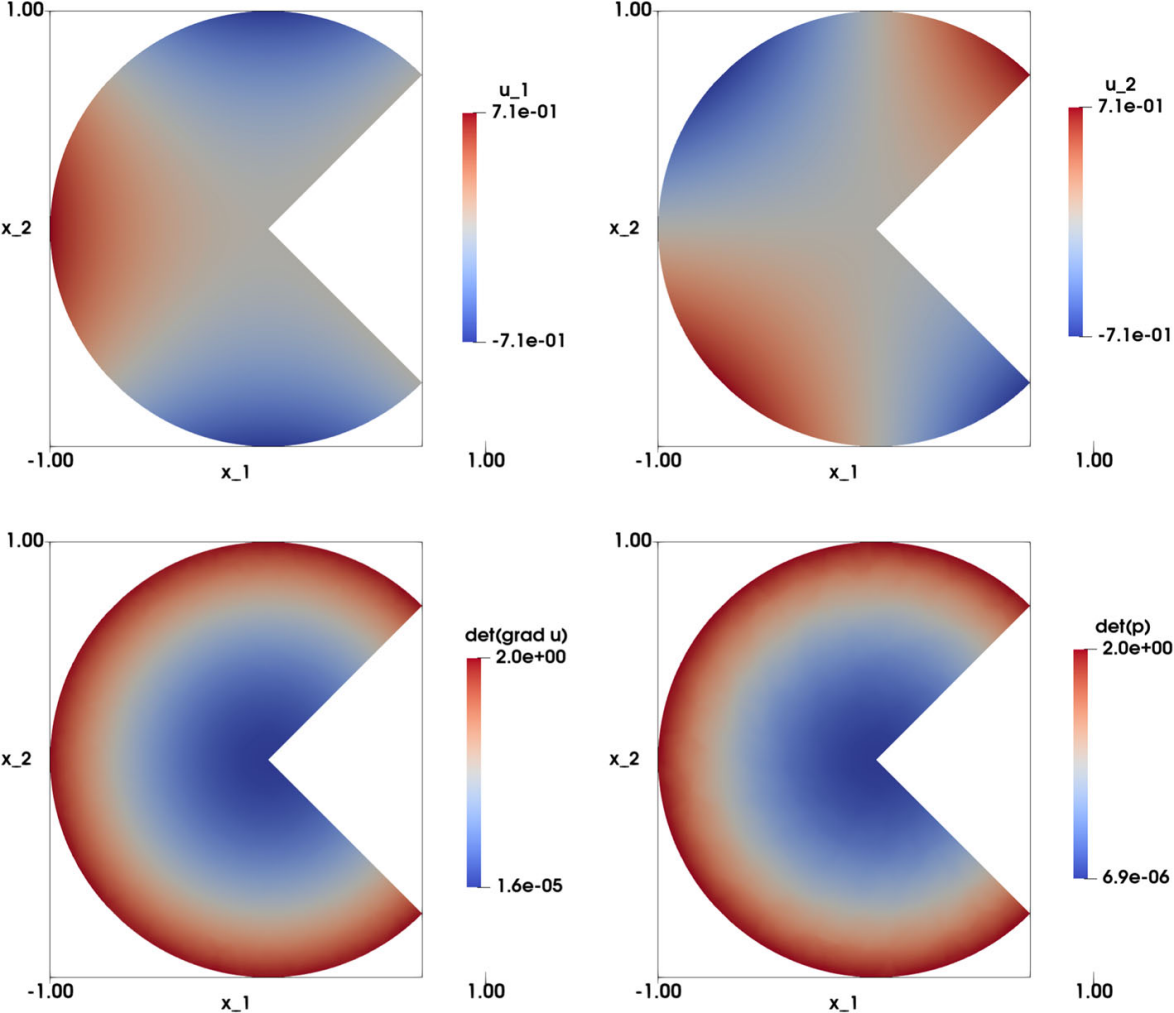
Smooth solution with radial right-hand side test case ($f(x_1,x_2)=2\left( x_1^2+x_2^2\right)$ and ${\mathbf {g}}(x_1,x_2)=\sqrt{2}\left( \frac{1}{2}\left( x_1^2-x_2^2\right) ,x_1x_2 \right) ^T$ on $\Gamma$). Top left: Numerical approximation of the solution of the component $u_{1,h}$. Top right: Numerical approximation of the solution of the component $u_{2,h}$. Bottom left: Numerical approximation of $\det \nabla {\mathbf {u}}_h$. Bottom right: Numerical approximation of $\det {\mathbf {p}}_h$. The results are obtained on a structured mesh of the pacman domain with $h=0.0252$

**Fig. 7:**
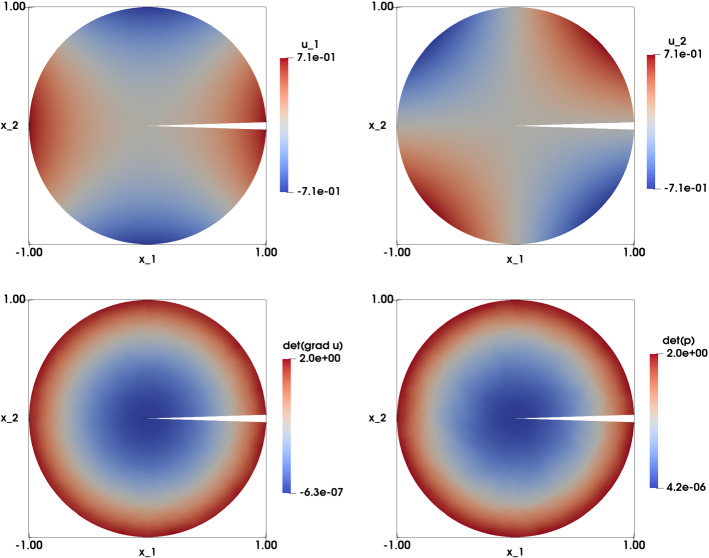
Smooth solution with radial right-hand side test case ($f(x_1,x_2)=2\left( x_1^2+x_2^2\right)$ and ${\mathbf {g}}(x_1,x_2)=\sqrt{2}\left( \frac{1}{2}\left( x_1^2-x_2^2\right) ,x_1x_2 \right) ^T$ on $\Gamma$). Top left: Numerical approximation of the solution of the component $u_{1,h}$. Top right: Numerical approximation of the solution of the component $u_{2,h}$. Middle left: Numerical approximation of $\det \nabla {\mathbf {u}}_h$. Middle right: Numerical approximation of $\det {\mathbf {p}}_h$. The results are obtained on a structured mesh of the cracked domain with $h=0.0486$

The numerical solution of (33) for both domains is illustrated in the top row of Figs. 6 and 7, respectively. In particular, we observe that the difference between $\det \nabla {\mathbf {u}}_h$ and $\det {\mathbf {p}}_h$ is vanishing, showing convergence of the least-squares method.

**Table 6:** Smooth solution with radial right-hand side test case. The case of the pacman disk with an unstructured mesh. ($f(x_1,x_2)=2\left( x_1^2+x_2^2\right)$ and ${\mathbf {g}}(x_1,x_2)=\sqrt{2}\left( \frac{1}{2}\left( x_1^2-x_2^2\right) ,x_1x_2 \right) ^T$ on $\Gamma$). Computational results include the mesh size *h*, the $L^2$ and $H^1$ error norms with the corresponding rates, the error $\left| \left| \nabla {\mathbf {u}}_h-{\mathbf {p}}_h\right| \right| _{L^2\left( \Omega \right) }$, the average value ${\bar{\lambda }}$ and its standard deviation ${\bar{\sigma }}$, and the number of iterations of the relaxation algorithm

*h*	$\vert \vert {\mathbf {u}}-{\mathbf {u}}_h\vert \vert _{L^2\left( \Omega \right) }$		$\vert {\mathbf {u}}-{\mathbf {u}}_h\vert _{H^1\left( \Omega \right) }$		$\vert \vert \nabla {\mathbf {u}}_h-{\mathbf {p}}_h\vert \vert _{L^2\left( \Omega \right) }$	${\bar{\lambda }}({\bar{\sigma }})$	iter
0.0747	3.24e$-$03		1.51e$-$01		7.68e$-$02	3.43e$-$03(0.045)	19
0.0503	8.65e$-$04	1.90	7.59e$-$02	1.00	3.90e$-$02	1.47e$-$03(0.025)	29
0.0252	2.26e$-$04	1.94	3.80e$-$02	1.00	1.95e$-$02	7.16e$-$04(0.013)	59
0.0126	6.67e$-$05	1.76	1.90e$-$02	1.00	9.73e$-$03	2.99e$-$04(0.007)	119

**Table 7:** Smooth solution with radial right-hand side test case. The case of the cracked disk with an unstructured mesh. ($f(x_1,x_2)=2\left( x_1^2+x_2^2\right)$ and ${\mathbf {g}}(x_1,x_2)=\sqrt{2}\left( \frac{1}{2}\left( x_1^2-x_2^2\right) ,x_1x_2 \right) ^T$ on $\Gamma$). Computational results include the mesh size *h*, the $L^2$ and $H^1$ error norms with the corresponding rates, the error $\left| \left| \nabla {\mathbf {u}}_h-{\mathbf {p}}_h\right| \right| _{L^2\left( \Omega \right) }$, the average value ${\bar{\lambda }}$ and its standard deviation ${\bar{\sigma }}$, and the number of iterations of the relaxation algorithm

*h*	$\vert \vert {\mathbf {u}}-{\mathbf {u}}_h\vert \vert _{L^2\left( \Omega \right) }$		$\vert {\mathbf {u}}-{\mathbf {u}}_h\vert _{H^1\left( \Omega \right) }$		$\vert \vert \nabla {\mathbf {u}}_h-{\mathbf {p}}_h\vert \vert _{L^2\left( \Omega \right) }$	${\bar{\lambda }}({\bar{\sigma }})$	iter
0.1525	1.42e$-$02		3.44e$-$01		1.81e$-$01	1.89e$-$02(0.101)	19
0.0971	3.98e$-$03	1.83	1.72e$-$01	1.00	9.15e$-$02	1.11e$-$02(0.056)	19
0.0486	1.12e$-$03	1.83	8.63e$-$02	1.00	4.53e$-$02	5.62e$-$03(0.030)	39
0.0243	3.33e$-$04	1.75	4.32e$-$02	1.00	2.23e$-$02	2.89e$-$03(0.016)	69

### Nonsmooth Right-Hand Side with a Line Singularity

Let us now consider $\Omega$ the unit disk, and a non-smooth right hand side with a singularity (jump) along a line in $\Omega$, and given by:

34 $$\begin{aligned} f\left( {\mathbf {x}}\right) ={\left\{ \begin{array}{ll} 0.1 \,\ \text {if } x_1 \le 0,\\ 1.9 \,\ \text {if } x_1 > 0 . \end{array}\right. } \end{aligned}$$

Note that *f* satisfies the necessary condition $\displaystyle \int _\Omega f=\text {measure}\left( \Omega \right)$. On the boundary, we impose ${\mathbf {g}}({\mathbf {x}})={\mathbf {x}}$. This problem has no known exact solution to the best of our knowledge.

Table 8 shows results for $\varepsilon =0$ and $\varepsilon =h^2$ for the disk structured meshes. We observe that the $L^2$ error between the numerical solution $\nabla {\mathbf {u}}_h$ and the auxiliary variable ${\mathbf {p}}_h$ is of the order of $O\left( h\right)$ for both values of $\varepsilon$, although it is more accurate for $\varepsilon =0$. This confirms that the accuracy of the method improves when *h* tends to zero. The same observations can be made for ${\bar{\lambda }}$ and ${\bar{\sigma }}$. The approximation ${\bar{\lambda }}$ decreases when *h* tends to zero and, more importantly, the same holds for ${\bar{\sigma }}$; this shows that the variability of those values across all triangles tends to zero, meaning the overall method accuracy increases. In addition, the iterations of the relaxation algorithm are reaching the limit of stopping criterion for $\varepsilon =0$; on the other hand, for $\varepsilon =h^2$, the iterations are well controlled. This shows that there are cases where the $\varepsilon$-regularization helps the convergence of the algorithm.

**Table 8:** Nonsmooth right-hand side with a line singularity. Results for $\varepsilon =0$ and $\varepsilon =h^2$. Computational results include the mesh size *h*, the error $\left| \left| \nabla {\mathbf {u}}_h-{\mathbf {p}}_h\right| \right| _{L^2\left( \Omega \right) }$, the average value ${\bar{\lambda }}$ and its standard deviation ${\bar{\sigma }}$, and the number of iterations of the relaxation algorithm. The case of the unit disk with a structured mesh

*h*	$\vert \vert \nabla {\mathbf {u}}_h-{\mathbf {p}}_h\vert \vert _{L^2\left( \Omega \right) }$	${\bar{\lambda }}({\bar{\sigma }})$	iter
$$\varepsilon =0$$			
0.0831	3.61e$-$02	7.27e$-$04(0.007)	999
0.0421	1.77e$-$02	1.81e$-$04(0.005)	999
0.0209	1.15e$-$02	$-$1.52e$-$05(0.003)	999
0.0104	8.65e$-$03	$-$1.40e$-$05(0.002)	999
$$\varepsilon =h^2$$			
0.0831	4.68e$-$01	$-$1.31e$-$01(0.166)	189
0.0421	2.69e$-$01	$-$3.36e$-$02(0.090)	129
0.0209	1.73e$-$01	$-$8.84e$-$03(0.059)	269
0.0104	1.14e$-$01	$-$2.42e$-$03(0.041)	779

**Fig. 8:**
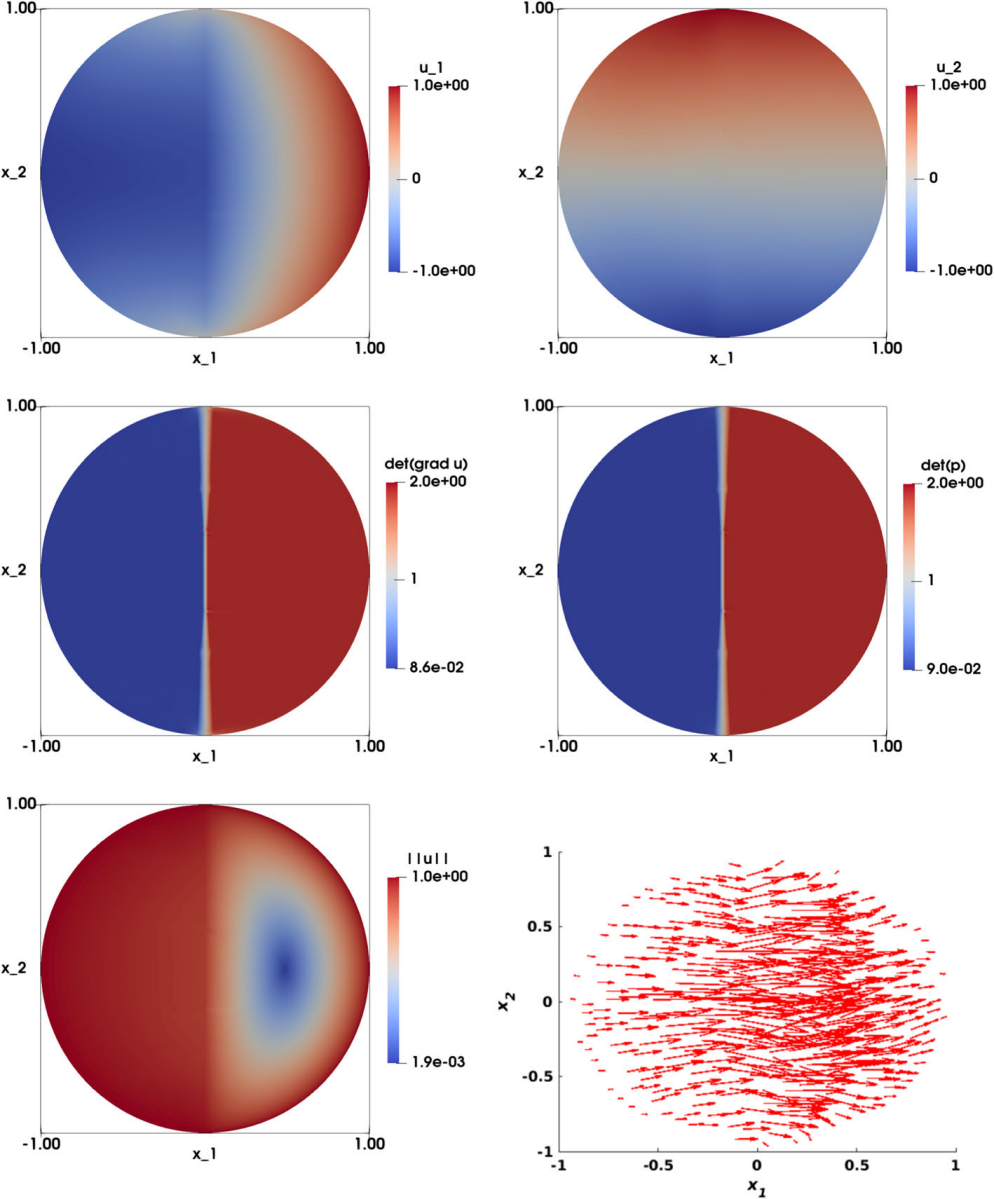
Nonsmooth right-hand side with a line singularity. Top left: Numerical approximation of the solution of the component $u_{1,h}$. Top right: Numerical approximation of the solution of the component $u_{2,h}$. Middle left: Numerical approximation of $\det \nabla {\mathbf {u}}_h$. Middle right: Numerical approximation of $\det {\mathbf {p}}_h$. Bottom left: Visualization of $\left| \left| {\mathbf {u}}_h\right| \right|$. Bottom right: Visualization of the vector field ${\mathbf {u}}_h$. The results are obtained on a structured mesh of the unit disk with $h=0209$ and $\varepsilon =0$

**Fig. 9:**
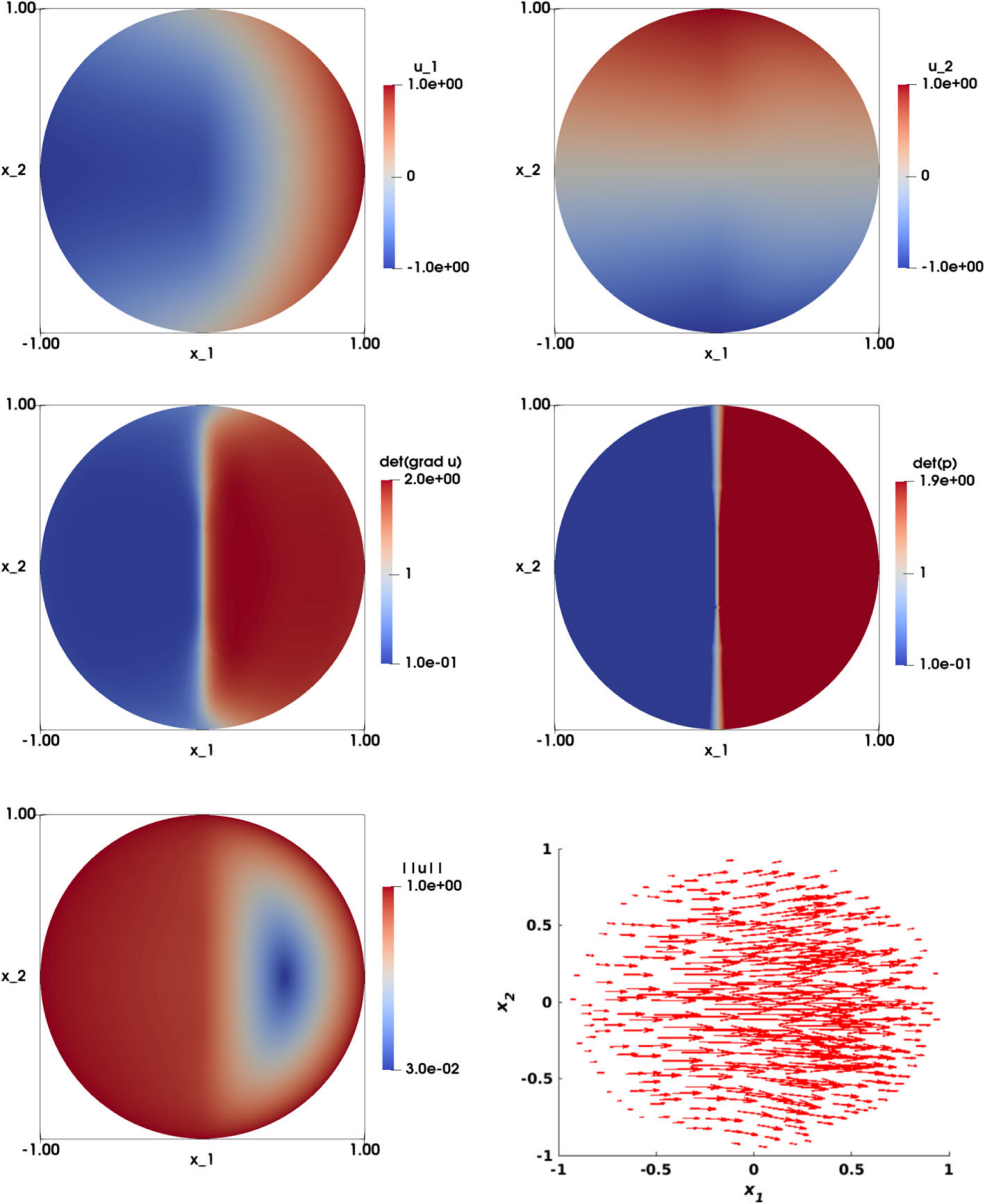
Nonsmooth right-hand side with a line singularity. Top left: Numerical approximation of the solution of the component $u_{1,h}$. Top right: Numerical approximation of the solution of the component $u_{2,h}$. Middle left: Numerical approximation of $\det \nabla {\mathbf {u}}_h$. Middle right: Numerical approximation of $\det {\mathbf {p}}_h$. Bottom left: Visualization of $\left| \left| {\mathbf {u}}_h\right| \right|$. Bottom right: Visualization of the vector field ${\mathbf {u}}_h$. The results are obtained on a structured mesh of the unit disk with $h=0209$ and $\varepsilon =h^2$

The numerical approximation of the solution for $\varepsilon =0$ is illustrated in Fig. 8. A close inspection shows that $u_{1,h}$ (top left) is discontinuous across $x_2=0$, as expected, while $u_{2,h}$ (top right) remains smooth. The numerical approximation of $\det \nabla {\mathbf {u}}_h$ and $\det {\mathbf {p}}_h$ are displayed in the second row and are identical. The numerical approximation of the solution for $\varepsilon =h^2$ is illustrated in Fig. 9. Both components look smooth. The second row shows the numerical approximations $\det \nabla {\mathbf {u}}_h$ and $\det {\mathbf {p}}_h$, which look different.

**Fig. 10:**
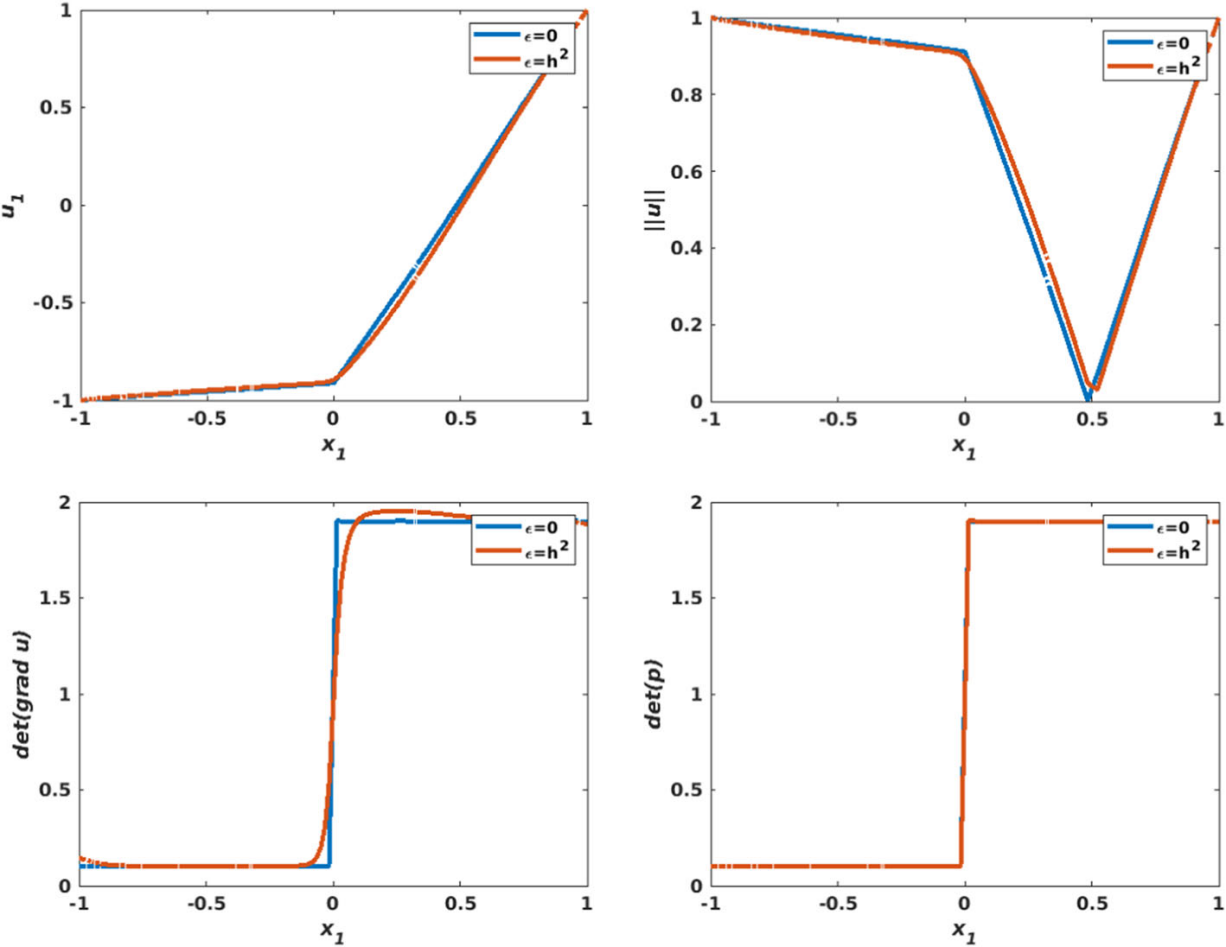
Nonsmooth right-hand side with a line singularity. Comparison plot between the approximations obtained with $\varepsilon =0$ and $\varepsilon =h^2$ respectively. Computation on a structured mesh with $h=0.0209$. Data is extracted along the line $x_2=0$. Top left : $u_{1,h}$ versus $x_1$. Top right: $\left| \left| {\mathbf {u}}_h\right| \right|$ vs $x_1$. Bottom left: $\det \nabla {\mathbf {u}}_h$ vs $x_1$. Bottom right: $\det {\mathbf {p}}_h$ vs $x_1$. The results are obtained on a structured mesh of the unit disk with $h=0.209$ and $\varepsilon =h^2$

Figure 10 illustrates a comparison between the two choices of $\varepsilon$, along the cutting line $x_2=0$. On the top row, – $u_{1,h}$ (left) and $\left| \left| {\mathbf {u}}_h\right| \right|$ (right)– a smoothing effect is observed when $\varepsilon > 0$ at the discontinuity point. On the bottom row, we observe an overshoot of the approximation $\det \nabla {\mathbf {u}}_h$ (left), while the approximation $\det {\mathbf {p}}_h$ (right) shows a solution that is independent of the choice of $\varepsilon$.

### Nonsmooth Problem Involving a Dirac Delta Function

Let us consider the unit disk $\Omega$, and a Dirac delta function $f(x_1,x_2)=\pi \delta _{(0,0)}$ for the right-hand side, and ${\mathbf {g}}(x_1,x_2)=(x_1,x_2)^T$ for the boundary conditions. One exact solution of the problem is, in this case,

35 $$\begin{aligned} {\mathbf {u}}\left( {\mathbf {x}}\right) =\frac{{\mathbf {x}}}{\vert \vert {\mathbf {x}}\vert \vert _2}. \end{aligned}$$

In order to apply our methodology, we approximate *f* by $f_\eta$ defined by

$$\begin{aligned} f_\eta \left( {\mathbf {x}}\right) =\frac{\eta ^2}{\left( \eta ^2+\left| \left| {\mathbf {x}}\right| \right| _2^2\right) ^2}\quad , \end{aligned}$$

where $\eta >0$ is a small positive value, see [1, 40]. When $\eta$ tends to 0, the approximate function $f_{\eta }$ converges to *f*. Note that $f_\eta$ satisfies

$$\begin{aligned} \int _\Omega f_\eta d{\mathbf {x}}=\frac{\pi }{\eta +1}, \end{aligned}$$

and also tends to the measure of $\Omega$ when $\eta$ tends to 0 (necessary condition). The modified problem then reads as

36 $$\begin{aligned} \left\{ \begin{array}{ll} \det \nabla {\mathbf {u}}_\eta ({\mathbf {x}}) = f_\eta ({\mathbf {x}}) &{}\text {in}~~\Omega ,\\ \\ {\mathbf {u}}_{\eta } \left( {\mathbf {x}}\right) ={\mathbf {x}}&{}\text {on}~~\Gamma , \end{array}\right. \end{aligned}$$

and the exact solution is

37 $$\begin{aligned} {\mathbf {u}}_\eta ({\mathbf {x}})= {\mathbf {x}}\sqrt{\frac{1+\eta ^2}{\eta ^2+\left| \left| {\mathbf {x}}\right| \right| _2^2}}. \end{aligned}$$

We can show that $\lim _{\eta \rightarrow 0} {\mathbf {u}}_\eta ={\mathbf {u}}$.

We will examine the approximation of the solution for various values of $\eta ,\varepsilon$ and *h*. Table 9 illustrates the computation of numerical approximations for different $\eta$ and *h*, and for an unstructured mesh on a unit disk. For the values of $\eta$ considered ($\eta \in \{1/8,1/16,1/32,1/64\}$), the error in $L^2$ norm decreases with an order equal or larger than *O*(*h*). The smaller the value of $\eta$, the less smooth the problem, and the smaller the convergence order of our algorithm. Indeed small values of $\eta$ are associated with solutions with large gradients, which would require a finer mesh in order for the algorithm to converge.

The same convergence behavior is observed for $\vert \vert \nabla {\mathbf {u}}_h-{\mathbf {p}}_h\vert \vert _{L^2\left( \Omega \right) }$, and for the estimates of ${\bar{\lambda }}$ and ${\bar{\sigma }}$. Note that the variability (symbolized by ${\bar{\sigma }}$) is decreasing when *h* tends to zero. Note also that, for large values of $\eta$, the necessary condition $\int _\Omega f_\eta = \pi$ is not satisfied, which impacts the convergence of the algorithm.

**Table 9:** Nonsmooth problem involving a Dirac delta function, with $f({\mathbf {x}})=\frac{\eta ^2}{\left( \eta ^2+\left| \left| {\mathbf {x}}\right| \right| _2^2\right) ^2}$ and ${\mathbf {g}} ({\mathbf {x}})={\mathbf {x}}\sqrt{\frac{1+\eta ^2}{\eta ^2+\left| \left| {\mathbf {x}}\right| \right| _2^2}}$. Convergence results for various $\eta$. Computational results include the mesh size *h*, the errors $\vert \vert {\mathbf {u}}-{\mathbf {u}}_h\vert \vert _{L^2\left( \Omega \right) }$ and $\left| \left| \nabla {\mathbf {u}}_h-{\mathbf {p}}_h\right| \right| _{L^2\left( \Omega \right) }$, the average value ${\bar{\lambda }}$ and its standard deviation ${\bar{\sigma }}$, and the number of iterations of the relaxation algorithm. The case of the unit disk with a structured mesh

*h*	$\vert \vert {\mathbf {u}}-{\mathbf {u}}_h\vert \vert _{L^2\left( \Omega \right) }$		$\vert \vert \nabla {\mathbf {u}}_h-{\mathbf {p}}_h\vert \vert _{L^2\left( \Omega \right) }$	${\bar{\lambda }}({\bar{\sigma }})$	iter
$$\eta =1/4$$					
0.0831	1.69e$-$01		5.69e$-$01	$-$4.94e$-$02(0.178)	59
0.0421	5.80e$-$02	1.54	2.01e$-$01	$-$2.87e$-$02(0.057)	99
0.0209	2.33e$-$02	1.31	8.55e$-$02	$-$2.16e$-$02(0.019)	239
0.0104	1.31e$-$02	0.83	6.01e$-$02	$-$1.87e$-$02(0.006)	999
$$\eta =1/8$$					
0.0831	2.13e$-$01		9.20e$-$01	$-$1.09e$-$01(0.181)	119
0.0421	7.73e$-$02	1.46	4.19e$-$01	$-$4.60e$-$02(0.069)	39
0.0209	2.66e$-$02	1.54	1.64e$-$01	$-$1.79e$-$02(0.026)	299
0.0104	9.71e$-$03	1.45	5.66e$-$02	$-$7.54e$-$03(0.009)	999
$$\eta =1/16$$					
0.0831	2.68e$-$01		1.43e+00	$-$2.08e$-$01(0.177)	359
0.0421	1.14e$-$01	1.23	8.40e$-$01	$-$9.08e$-$02(0.083)	249
0.0209	4.37e$-$02	1.39	4.11e$-$01	$-$3.37e$-$02(0.035)	329
0.0104	1.47e$-$02	1.57	1.61e$-$01	$-$1.09e$-$02(0.013)	999
$$\eta =1/32$$					
0.0831	3.21e$-$01		2.03e+00	$-$3.04e$-$01(0.167)	999
0.0421	1.61e$-$01	0.99	1.38e+00	$-$1.46e$-$01(0.092)	79
0.0209	7.33e$-$02	1.14	8.37e$-$01	$-$6.16e$-$02(0.044)	589
0.0104	2.85e$-$02	1.36	4.11e$-$01	$-$2.26e$-$02(0.018)	379
$$\eta =1/64$$					
0.0831	3.68e$-$01		2.39e+00	$-$3.88e$-$01(0.153)	999
0.0421	2.09e$-$01	0.81	1.99e+00	$-$2.05e$-$01(0.097)	229
0.0209	1.13e$-$01	0.89	1.38e+00	$-$1.00e$-$01(0.051)	999
0.0104	5.29e$-$02	1.09	8.36e$-$01	$-$4.39e$-$02(0.023)	999

Table 10 illustrates numerical results for a fixed $h=0.0209$ and various $\eta$. We observe that, when $\eta$ tends to zero, the solutions presents a stronger singularity, and all indicators in the table are increasing.

**Table 10:** Nonsmooth problem involving a Dirac delta function, with $f({\mathbf {x}})=\frac{\eta ^2}{\left( \eta ^2+\left| \left| {\mathbf {x}}\right| \right| _2^2\right) ^2}$ and ${\mathbf {g}} ({\mathbf {x}})={\mathbf {x}}\sqrt{\frac{1+\eta ^2}{\eta ^2+\left| \left| {\mathbf {x}}\right| \right| _2^2}}$. Convergence results for various $\eta$ and fixed mesh ($h=0.0209$). Computational results include $\eta$, the errors $\vert \vert {\mathbf {u}}-{\mathbf {u}}_h\vert \vert _{L^2\left( \Omega \right) }$ and $\left| \left| \nabla {\mathbf {u}}_h-{\mathbf {p}}_h\right| \right| _{L^2\left( \Omega \right) }$, the average value ${\bar{\lambda }}$ and its standard deviation ${\bar{\sigma }}$, and the number of iterations of the relaxation algorithm. The case of the unit disk with a structured mesh

$\eta$	$\vert \vert {\mathbf {u}}-{\mathbf {u}}_h\vert \vert _{L^2\left( \Omega \right) }$	$\vert \vert \nabla {\mathbf {u}}_h-{\mathbf {p}}_h\vert \vert _{L^2\left( \Omega \right) }$	${\bar{\lambda }}({\bar{\sigma }})$	iter
$$h=0.0209$$				
1/4	2.33e$-$02	8.55e$-$02	$-$2.16e$-$02(0.019)	239
1/8	2.66e$-$02	1.64e$-$01	$-$1.79e$-$02(0.026)	299
1/16	4.37e$-$02	4.11e$-$01	$-$3.37e$-$02(0.035)	329
1/32	7.33e$-$02	8.37e$-$01	$-$6.16e$-$02(0.044)	589
1/64	1.13e$-$01	1.38e+00	$-$1.00e$-$01(0.051)	999

Table 11 represents the $L^{\infty }$ norm of $f_\eta$, $\det {\mathbf {p}}_h$ and $\det \nabla {\mathbf {u}}_h$ for various values of $\eta$. We observe that the values of $\det {\mathbf {p}}_h$ and $f_\eta$ have the same order of magnitude, for $\eta =\{1/4,1/8,1/16,1/32\}$. However, the last row of Table 11 exhibits large differences, which is sign of the need of a finer mesh. Table 12 shows, for two fixed values of $\eta$, convergence results when *h* and $\varepsilon$ vary. We observe that, for $\varepsilon =\{0,h^2\}$, the numerical solution converges in $L^2$-norm with a rate $O\left( h\right)$ (or better). Moreover, $\vert \vert \nabla {\mathbf {u}}_h-{\mathbf {p}}_h\vert \vert _{L^2\left( \Omega \right) }$ decreases with an order of $O\left( h\right)$. Similar effects occur for ${\bar{\lambda }}$ and ${\bar{\sigma }}$. The number of iterations of the relaxation algorithm reaches its maximal number when $\varepsilon =0$.

**Table 11:** Nonsmooth problem involving a Dirac delta function, with $f({\mathbf {x}})=\frac{\eta ^2}{\left( \eta ^2+\left| \left| {\mathbf {x}}\right| \right| _2^2\right) ^2}$ and ${\mathbf {g}} ({\mathbf {x}})={\mathbf {x}}\sqrt{\frac{1+\eta ^2}{\eta ^2+\left| \left| {\mathbf {x}}\right| \right| _2^2}}$. Convergence results for various $\eta$ and a fixed mesh ($h=0.0209$). Computational results include $\eta$, and the values of the $L^\infty$-norm of $\det {\mathbf {p}}_h$, $\det \nabla {\mathbf {u}}_h,$ and $f_\eta$. The case of the unit disk with a structured mesh

$\eta$	$\vert \vert \det {\mathbf {p}}_h\vert \vert _{L^{\infty }\left( \Omega \right) }$	$\vert \vert \det \nabla {\mathbf {u}}_h\vert \vert _{L^{\infty }\left( \Omega \right) }$	$\vert \vert f_\eta \vert \vert _{L^{\infty }\left( \Omega \right) }$
$$h=0.0209$$			
1/4	15.99	14.81	16
1/8	63.88	46.97	64
1/16	254.23	119.25	256
1/32	996.13	218.06	1024
1/64	3676.18	280.17	4096

**Table 12:** Nonsmooth problem involving a Dirac delta function, with $f({\mathbf {x}})=\frac{\eta ^2}{\left( \eta ^2+\left| \left| {\mathbf {x}}\right| \right| _2^2\right) ^2}$ and ${\mathbf {g}} ({\mathbf {x}})={\mathbf {x}}\sqrt{\frac{1+\eta ^2}{\eta ^2+\left| \left| {\mathbf {x}}\right| \right| _2^2}}$. Convergence results for various $\varepsilon$ ($\eta = 1/32$). Computational results include the mesh size *h*, the errors $\vert \vert {\mathbf {u}}-{\mathbf {u}}_h\vert \vert _{L^2\left( \Omega \right) }$ and $\left| \left| \nabla {\mathbf {u}}_h-{\mathbf {p}}_h\right| \right| _{L^2\left( \Omega \right) }$, the average value ${\bar{\lambda }}$ and its standard deviation ${\bar{\sigma }}$, and the number of iterations of the relaxation algorithm. The case of the unit disk with a structured mesh

*h*	$\vert \vert {\mathbf {u}}-{\mathbf {u}}_h\vert \vert _{L^2\left( \Omega \right) }$		$\vert \vert \nabla {\mathbf {u}}_h-{\mathbf {p}}_h\vert \vert _{L^2\left( \Omega \right) }$	${\bar{\lambda }}({\bar{\sigma }})$	iter
$$\eta =1/32,\ \varepsilon =0$$					
0.0831	7.90e$-$01		4.98e$-$01	9.51e$-$03(0.042)	999
0.0421	2.14e$-$01	1.89	3.08e$-$01	$-$1.24e$-$02(0.026)	999
0.0209	9.60e$-$02	1.16	1.65e$-$01	$-$7.98e$-$04(0.013)	999
0.0104	4.87e$-$02	0.98	9.73e$-$02	$-$2.04e$-$04(0.007)	999
$$\eta =1/32,\ \varepsilon =h^2$$					
0.0831	3.21e$-$01		2.03e+00	$-$3.04e$-$01(0.167)	999
0.0421	1.61e$-$01	0.99	1.38e+00	$-$1.46e$-$01(0.092)	79
0.0209	7.33e$-$02	1.14	8.37e$-$01	$-$6.16e$-$02(0.044)	589
0.0104	2.85e$-$02	1.36	4.11e$-$01	$-$2.26e$-$02(0.018)	379

**Fig. 11:**
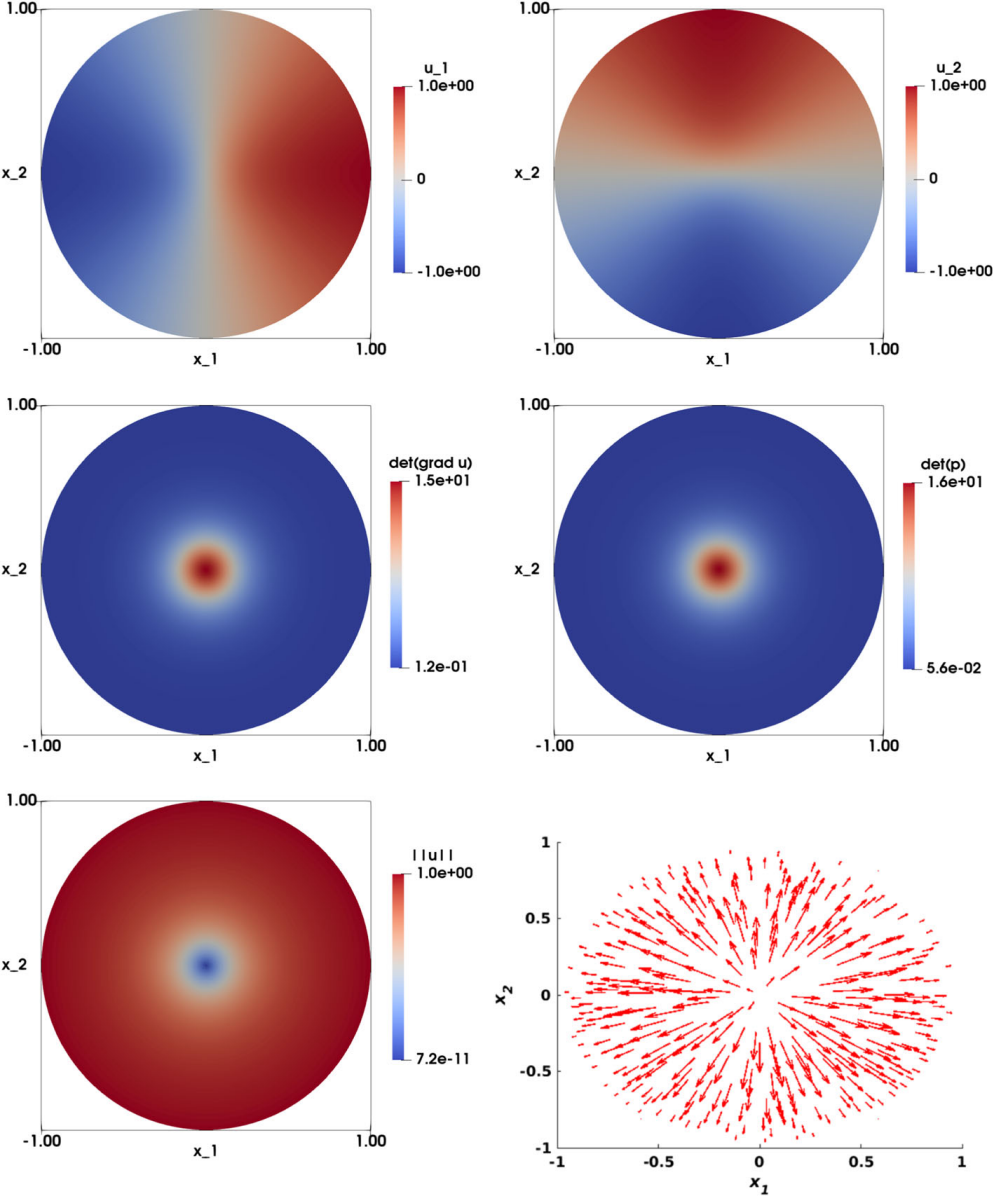
Non-smooth problem involving a Dirac delta function ($f({\mathbf {x}})=\frac{\eta ^2}{\left( \eta ^2+\left| \left| {\mathbf {x}}\right| \right| _2^2\right) ^2}$, ${\mathbf {g}}({\mathbf {x}})={\mathbf {x}}\sqrt{\frac{1+\eta ^2}{\eta ^2+\left| \left| {\mathbf {x}}\right| \right| _2^2}}$, $\eta =1/4$). Top left: Numerical approximation of the solution of the component $u_{1,h}$. Top right: Numerical approximation of the solution of the component $u_{2,h}$. Middle left: Numerical approximation of $\det \nabla {\mathbf {u}}_h$. Middle right: Numerical approximation of $\det {\mathbf {p}}_h$. Bottom left: Visualization of $\left| \left| {\mathbf {u}}_h\right| \right|$. Bottom right: Visualization of the vector field ${\mathbf {u}}_h$. The results are obtained on structured mesh of the unit disk with $h=0.0209$ and $\varepsilon =h^2$

**Fig. 12:**
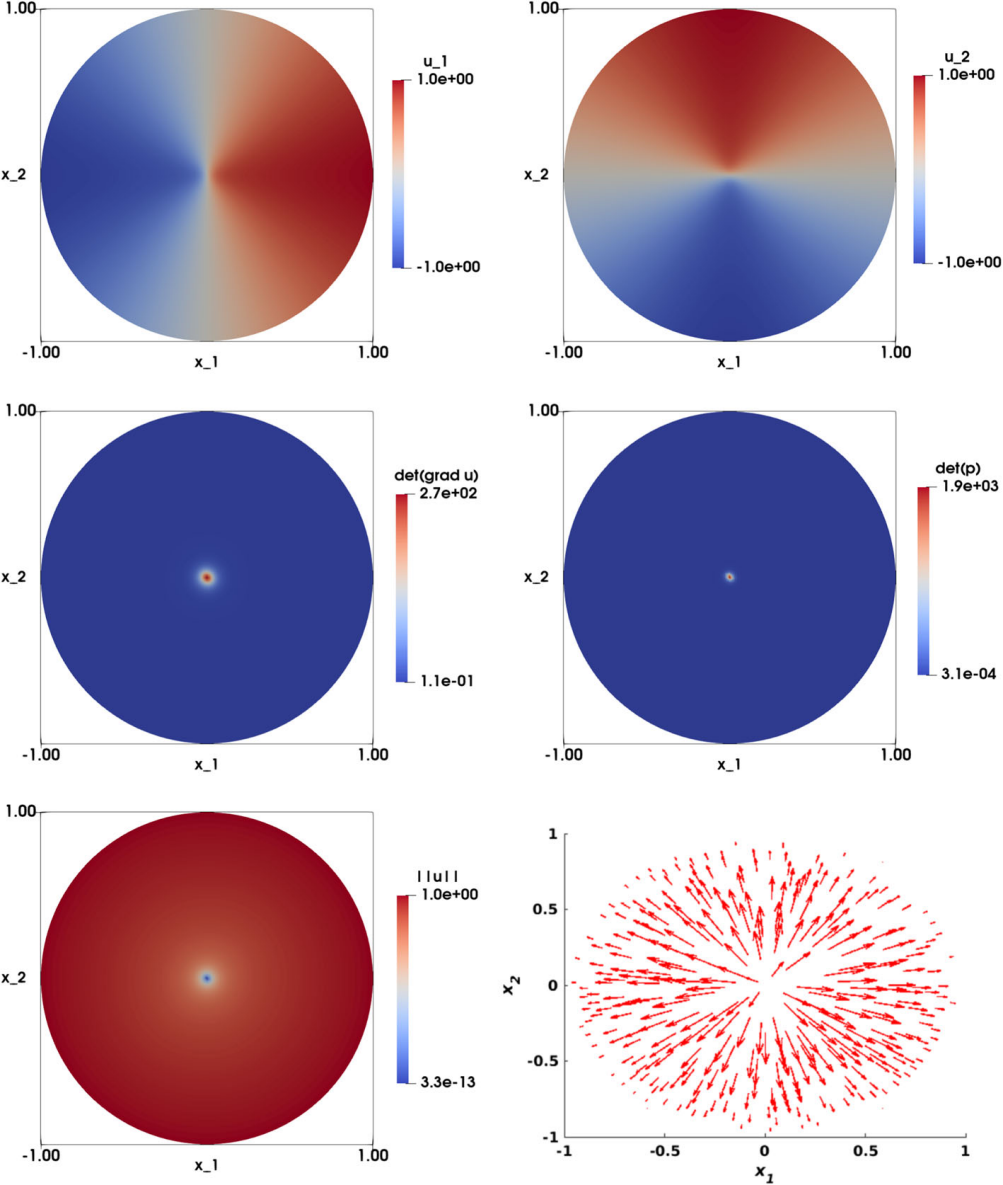
Non-smooth problem involving a Dirac delta function ($f({\mathbf {x}})=\frac{\eta ^2}{\left( \eta ^2+\left| \left| {\mathbf {x}}\right| \right| _2^2\right) ^2}$, ${\mathbf {g}}({\mathbf {x}})={\mathbf {x}}\sqrt{\frac{1+\eta ^2}{\eta ^2+\left| \left| {\mathbf {x}}\right| \right| _2^2}}$, $\eta =1/64$). Top left: Numerical approximation of the solution of the component $u_{1,h}$. Top right: Numerical approximation of the solution of the component $u_{2,h}$. Middle left: Numerical approximation of $\det \nabla {\mathbf {u}}_h$. Middle right: Numerical approximation of $\det {\mathbf {p}}_h$. Bottom left: Visualization of $\left| \left| {\mathbf {u}}_h\right| \right|$. Bottom right: Visualization of the vector field ${\mathbf {u}}_h$. The results are obtained on structured mesh of the unit disk with $h=0.0209$ and $\varepsilon =h^2$

Figures 11 and 12 illustrate the numerical approximation obtained for $\eta = 1/4$ and $\eta = 1/64$ respectively (with $\varepsilon = h^2$). We see that the solution of the two components $u_{1,h}$ and $u_{2,h}$ are smooth for $\eta$ sufficiently large ($\eta =\{1/4, 1/8\}$), and the singularity in (0, 0) is visible for $\eta$ smaller ($\eta =\{1/16, 1/32, 1/64\}$). The numerical approximations of $\det \nabla {\mathbf {u}}_h$ and $\det {\mathbf {p}}_h$ are illustrated in the second row of both figures, and shows that as $\eta$ gets smaller, the singularity become more prominent. The largest values of these determinants have been shown in Table 11.

**Fig. 13:**
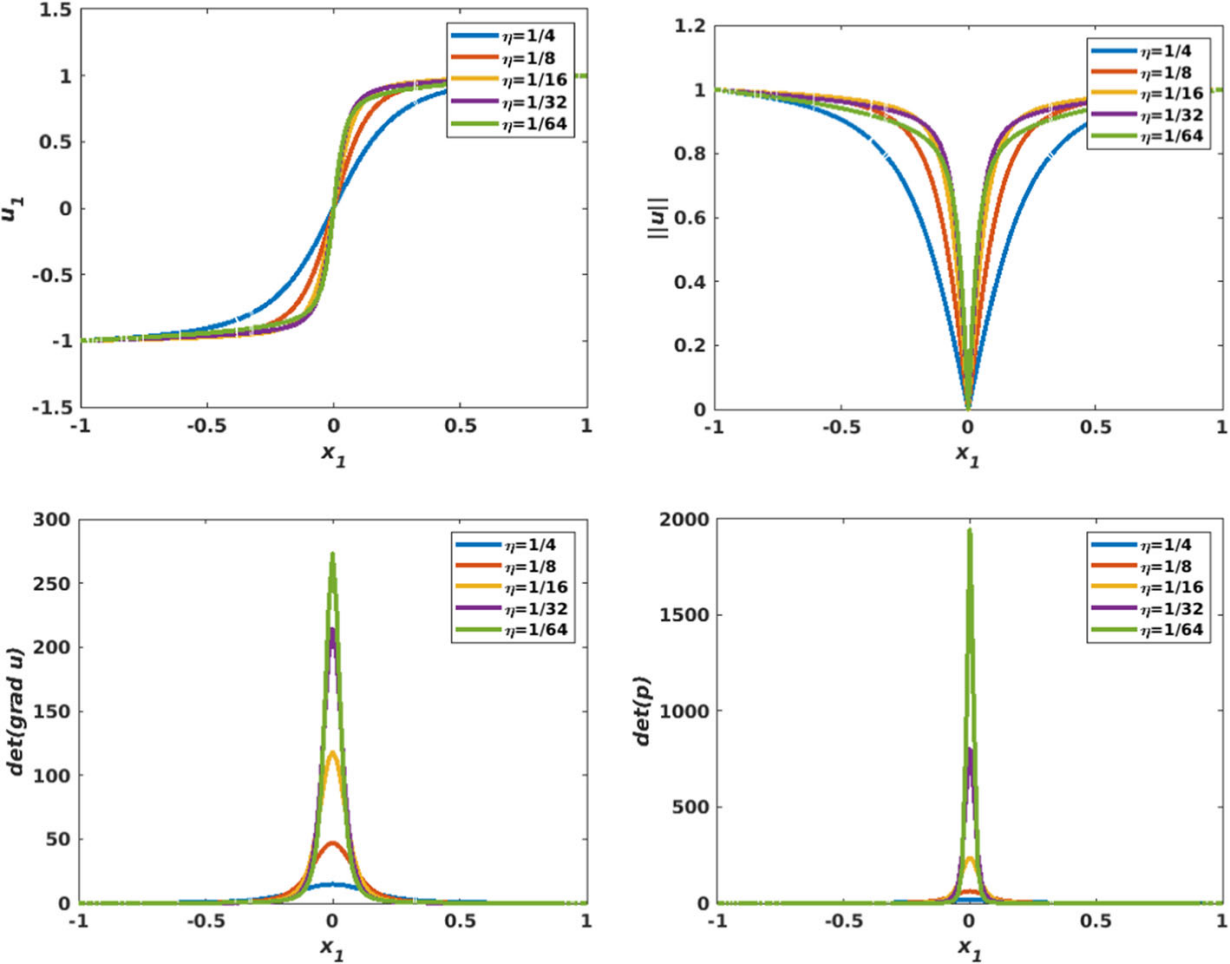
Non-smooth problem involving a Dirac delta function ($f({\mathbf {x}})=\frac{\eta ^2}{\left( \eta ^2+\left| \left| {\mathbf {x}}\right| \right| _2^2\right) ^2}$, ${\mathbf {g}}({\mathbf {x}})={\mathbf {x}}\sqrt{\frac{1+\eta ^2}{\eta ^2+\left| \left| {\mathbf {x}}\right| \right| _2^2}}$), with various values of the parameter $\eta$. Comparing profiles along the line $x_2=0$, for $u_{1,h}$ (top left), $\left| \left| {\mathbf {u}}_h\right| \right|$ (top right), $\det \nabla {\mathbf {u}}_h$ (bottom left), and $\det {\mathbf {p}}_h$ (bottom right). The results are obtained on structured mesh of the unit disk with $h=0.0209$

Figure 13 visualizes a comparison of the different figures associated with various values of $\eta$, with plots along the line $x_2=0$ (by symmetry). In the top row, both the approximation of $u_{1,h}$ (left) and $\left| \left| {\mathbf {u}}_h\right| \right|$ (right) are visualized. As $\eta$ gets smaller, the tangent line to the graph of $u_{1,h}$ at the singularity point (0, 0) becomes vertical, and the gradient undefined. This singularity point significantly appears on the plot of $\vert \vert {\mathbf {u}}_h\vert \vert$. Figure 13 (second row) shows $\det \nabla {\mathbf {u}}_h$ (left) and $\det {\mathbf {p}}_h$(right).

**Fig. 14:**
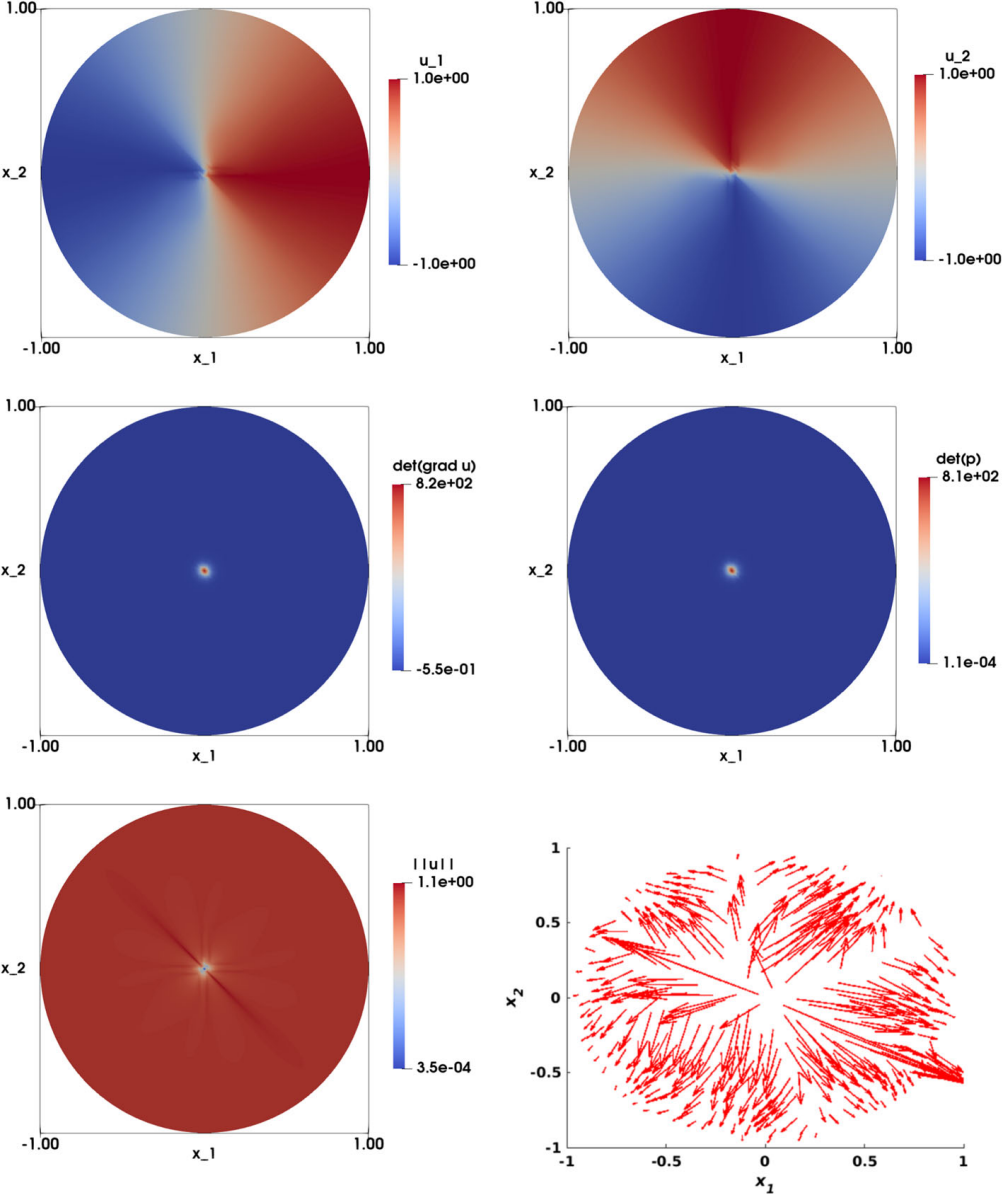
Non-smooth problem involving a Dirac delta function ($f({\mathbf {x}})=\frac{\eta ^2}{\left( \eta ^2+\left| \left| {\mathbf {x}}\right| \right| _2^2\right) ^2}$, ${\mathbf {g}}({\mathbf {x}})={\mathbf {x}}\sqrt{\frac{1+\eta ^2}{\eta ^2+\left| \left| {\mathbf {x}}\right| \right| _2^2}}$), with various values of the parameter $\eta$. Top left: Numerical approximation of the solution of the component $u_{1,h}$. Top right: Numerical approximation of the solution of the component $u_{2,h}$. Middle left: Numerical approximation of $\det \nabla {\mathbf {u}}_h$. Middle right: Numerical approximation of $\det {\mathbf {p}}_h$. Bottom left: Visualization of $\left| \left| {\mathbf {u}}_h\right| \right|$. Bottom right: Visualization of the vector field ${\mathbf {u}}_h$. The results are obtained on structured mesh of the unit disk with $h=0.0209$ and $\varepsilon =0$

**Fig. 15:**
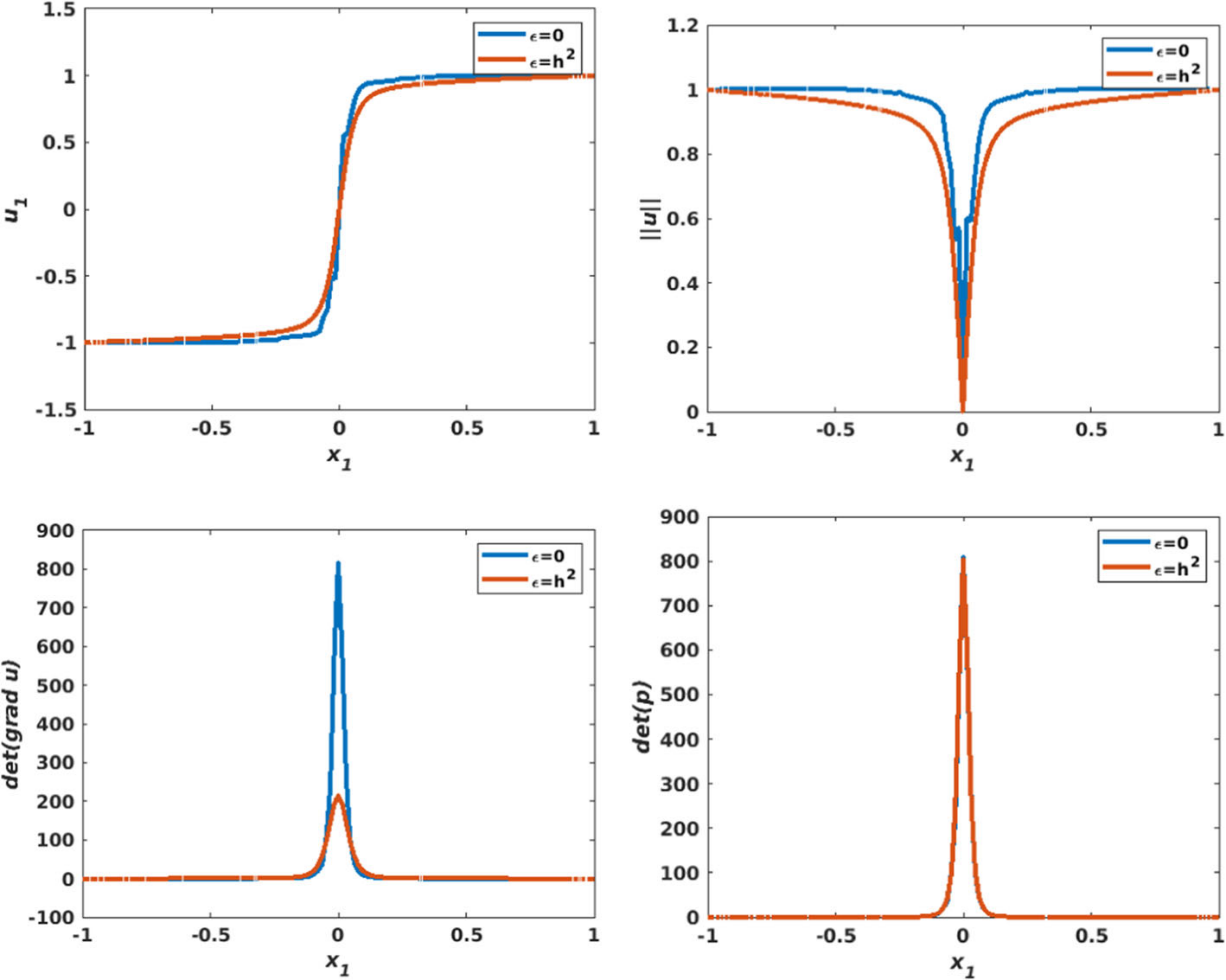
Non-smooth problem involving a Dirac delta function ($f({\mathbf {x}})=\frac{\eta ^2}{\left( \eta ^2+\left| \left| {\mathbf {x}}\right| \right| _2^2\right) ^2}$, ${\mathbf {g}}({\mathbf {x}})={\mathbf {x}}\sqrt{\frac{1+\eta ^2}{\eta ^2+\left| \left| {\mathbf {x}}\right| \right| _2^2}}$), with various values of the parameter $\eta$, with $\varepsilon =0$ and $\varepsilon =h^2$. Comparing profiles along the line $x_2=0$, for $u_{1,h}$ (top left), $\left| \left| {\mathbf {u}}_h\right| \right|$ (top right), $\det \nabla {\mathbf {u}}_h$ (bottom left), and $\det {\mathbf {p}}_h$ (bottom right). The results are obtained on structured mesh of the unit disk with $h=0.0209$

Figure 14 illustrates the numerical approximation of the solution when $\eta =1/32$, $\varepsilon =0$ and $h=0.0209$. The solutions of the two components $u_{1,h}$ and $u_{2,h}$ (first row) are non-smooth and the singularity at the origin is visible; this point also appears in the plots of $\det \nabla {\mathbf {u}}_h$ and $\det {\mathbf {p}}_h$ (second row). Figure 15 shows a comparison between the solution obtained with $\varepsilon =0$ and $\varepsilon =h^2$ ($\eta =1/32$, $h=0.0209$), by plotting the solution along the line $x_2=0$. We observe that the curves obtained when $\varepsilon =h^2$ have less oscillations and are smoother than those obtained when $\varepsilon =0$.

## Conclusions and Perspectives

A least-squares/relaxation finite element method has been advocated for the numerical solution of the prescribed Jacobian equation. The relaxation algorithm that decouples this least-squares problem into a sequence of local nonlinear problems and variational linear problems has shown optimal convergence orders for smooth problems. It has also proved to be robust in non-smooth cases, with nearly optimal convergence orders.

Generally speaking, the least-squares approach is as efficient as the augmented Lagrangian methodology introduced in [31], but without the required fine-tuning of the augmentation parameters, a well known drawback of ADMM algorithms.

## Data Availability

Enquiries about data availability should be directed to the authors.
